# Supplementary material for: Effects of plyometric training on jump, sprint, and change of direction performance in adolescent soccer player: A systematic review with meta-analysis
Source: PLoS One. 2025 Apr 29;20(4):e0319548. doi: 10.1371/journal.pone.0319548 (PMC12040276; doi:10.1371/journal.pone.0319548)
Supplement: S1 Checklist — (DOCX) [file pone.0319548.s003.docx]

| **Section and Topic** | **Item #** | **Checklist item** | **Location where item is reported** |
| --- | --- | --- | --- |
| **TITLE** | | |  |
| Title | 1 | Enhancement of Jump, Sprint, and Change of Direction Abilities: The Efficacy of Plyometric Training in Adolescent Soccer Training—A Systematic Review and Meta-Analysis | P1  (L1-L3) |
| **ABSTRACT** | | |  |
| Abstract | 2 | Background: Soccer, as one of the most popular sports globally, demands a high level of physical fitness from its participants. Particularly for adolescent soccer players, who are in a critical period of physical development, effective training methods are essential to enhance their sport Performance. Plyometric Training, known for improving lower limb explosiveness, has been proven effective for adult athletes, yet its impact on adolescent soccer players remains insufficiently and systematically assessed.  Objective: This study aims to systematically evaluate the comprehensive effects of plyometric training on adolescent soccer players' jumping, sprinting, and change-of-direction abilities, with the intention of providing an empirical basis for coaches and athletes to optimize training programs and enhance competitive performance.  Methods: Eligible randomized controlled trials were identified through searches of PubMed, Web of Science, Scopus, and SPORTDiscus databases. Study quality was assessed using the PEDro scale, and statistical analysis was conducted using Stata software to calculate the standardized mean difference (SMD) and 95% confidence intervals.  Results: A total of 20 studies, comprising 28 randomized controlled trials with a total sample size of 796 participants, were included. The results indicated that plyometric training significantly positively affected the jumping ability (SMD=0.76, 95%CI: 0.59, 0.93), sprinting ability (SMD=-0.45, 95%CI: -0.57, -0.32), and change-of-direction ability (SMD=-0.76, 95%CI: -1.04, -0.47) of adolescent soccer players.  Conclusion: Plyometric training is an effective training method to enhance the sport performance of adolescent soccer players. Compared to traditional soccer training, a training regimen incorporating PT significantly improves athletes' overall performance in jumping, sprinting, and change-of-direction abilities. Therefore, it is recommended that PT be included in the training of adolescent soccer players to promote their physical and athletic skill development.  Keywords: Plyometric Training; Adolescents; Soccer; Jumping. | P1  (L11-L36) |
| **INTRODUCTION** | | |  |
| Rationale | 3 | Soccer, one of the world's most beloved sports, draws countless enthusiastic participants into its fold[1]. Concurrently, to cater to the needs of various demographics, a multitude of soccer leagues have emerged, offering a platform for soccer enthusiasts to showcase their skills[2]. Despite the vast array of soccer players with differing levels of athletic prowess and age groups, they all share a common trait: the necessity for a solid foundation of physical fitness to exhibit commendable performance on the field[2].  Specifically, during a typical soccer match, players are often seen performing frequent jumps, sprints, and changes in direction, which are undoubtedly the most fundamental and common movements in soccer[3, 4]. Moreover, a team's average jumping height and sprinting ability are significantly correlated with their level of play and performance[2, 5]. High-level athletes tend to possess superior jumping, running, and change-of-direction abilities compared to their lower-level counterparts. Additionally, high-level professional league players or international players often exhibit higher levels of jumping, sprinting, and change-of-direction abilities during their adolescence compared to their peers[6]. Therefore, it is particularly essential to cultivate and develop these abilities during the adolescent phase for young soccer players[7].  Plyometric training, an effective method for enhancing lower limb explosiveness, is widely employed across various sports disciplines[8]. This training strategy ingeniously capitalizes on the Stretch-Shortening Cycle (SSC) [9], during which the muscle undergoes eccentric, isometric, and concentric contractions, effectively storing elastic potential energy[9]. When the muscle rapidly transitions from a lengthened state to a shortened state, this stored energy is released, enabling the muscle to generate immense force instantaneously, thereby significantly enhancing the athlete's explosive power[10, 11]. Meylan et al. [12] also confirmed the effectiveness of plyometric training in improving lower limb explosiveness in adolescent soccer players. Chen et al.[8] demonstrated that plyometric training positively influenced countermovement jumps (CMJ) and 20-meter sprint performance in adolescent soccer players of different developmental stages; however, their systematic review and meta-analysis only addressed CMJ and 20-meter sprint performance. | P1-P2  (L39-L67) |
| Objectives | 4 | this study aims to systematically evaluate the combined impact of plyometric training on adolescent soccer players' abilities in jumping, sprinting, and changing direction. By delving into the enhancement of these key athletic capabilities, this study not only seeks to reveal the direct effects of training interventions but also endeavors to provide an empirical basis for coaches and athletes to optimize training programs and enhance competitive performance. | P2  (L69-L75) |
| **METHODS** | | |  |
| Eligibility criteria | 5 | \| Category \| Inclusion criteria \| Exclusion Criteria \| \| --- \| --- \| --- \| \| Population（P） \| The study population consists exclusively of adolescent soccer players, with the age range defined as 10 to 18.99 years old. \| Participants who are not healthy or do not fall within the specified age range for adolescent soccer players. \| \| Intervention（I） \| Studies included should involve participants engaging in plyometric training either solely or in conjunction with their regular soccer training. \| Studies where participants undertake additional training regimens, such as resistance training or high-intensity interval training, alongside plyometric training. \| \| Comparator（C） \| The control group should only engage in soccer-specific training. \| Studies lacking a control group. \| \| Outcome（O） \| Outcome measures should include indicators of jumping, sprinting, or change-of-direction abilities (either in full or in part). \| Studies without baseline data or where the full text is inaccessible. \| \| Study design（S） \| Randomized controlled trials \| Cross-sectional studies, case studies. \| | P4  (L106) |
| Information sources | 6 | This study conducted a comprehensive search of four databases—PubMed, Web of Science (all databases), Scopus, and SPORTDiscus—from the inception of each database up to August 7, 2024. Boolean operators (OR, AND) were utilized in conjunction with a series of keywords, which were finalized based on literature reviews, meta-analyses, expert opinions, and the MeSH Database. The specific search string was as follows: ("Lower limb explosive strength" OR "Explosive strength" OR "explosive force" OR "Explosive power" OR "power" OR "Countermovement jump" OR "CMJ" OR "squat jump" OR "SJ" OR "standing long jump" OR "SLJ" OR "drop jump" OR "DJ" OR "sprint performance" OR "10m" OR "20m" OR "30m" OR "50m" OR "vertical jump" OR "VJ" OR "change of direction" OR "COD") AND ("plyometric" OR "plyometrics" OR "PT" OR "pliometrique" OR "entrainement pliometrique" OR "salto pliome´trico" OR "velocidad") AND ("Adolescent" OR "Adolescents" OR "Adolescence" OR "Teens" OR "Teen" OR "Teenagers" OR "Teenager" OR "Youth" OR "Youths" OR "Female Adolescent" OR "Female Adolescents" OR "Male Adolescent" OR "Male Adolescents" OR "Child" OR "Children") AND ("soccer"). | P2  (L78-L92) |
| Search strategy | 7 | \| Category \| Inclusion criteria \| Exclusion Criteria \| \| --- \| --- \| --- \| \| Population（P） \| The study population consists exclusively of adolescent soccer players, with the age range defined as 10 to 18.99 years old. \| Participants who are not healthy or do not fall within the specified age range for adolescent soccer players. \| \| Intervention（I） \| Studies included should involve participants engaging in plyometric training either solely or in conjunction with their regular soccer training. \| Studies where participants undertake additional training regimens, such as resistance training or high-intensity interval training, alongside plyometric training. \| \| Comparator（C） \| The control group should only engage in soccer-specific training. \| Studies lacking a control group. \| \| Outcome（O） \| Outcome measures should include indicators of jumping, sprinting, or change-of-direction abilities (either in full or in part). \| Studies without baseline data or where the full text is inaccessible. \| \| Study design（S） \| Randomized controlled trials \| Cross-sectional studies, case studies. \| | P4  (L106) |
| Selection process | 8 | 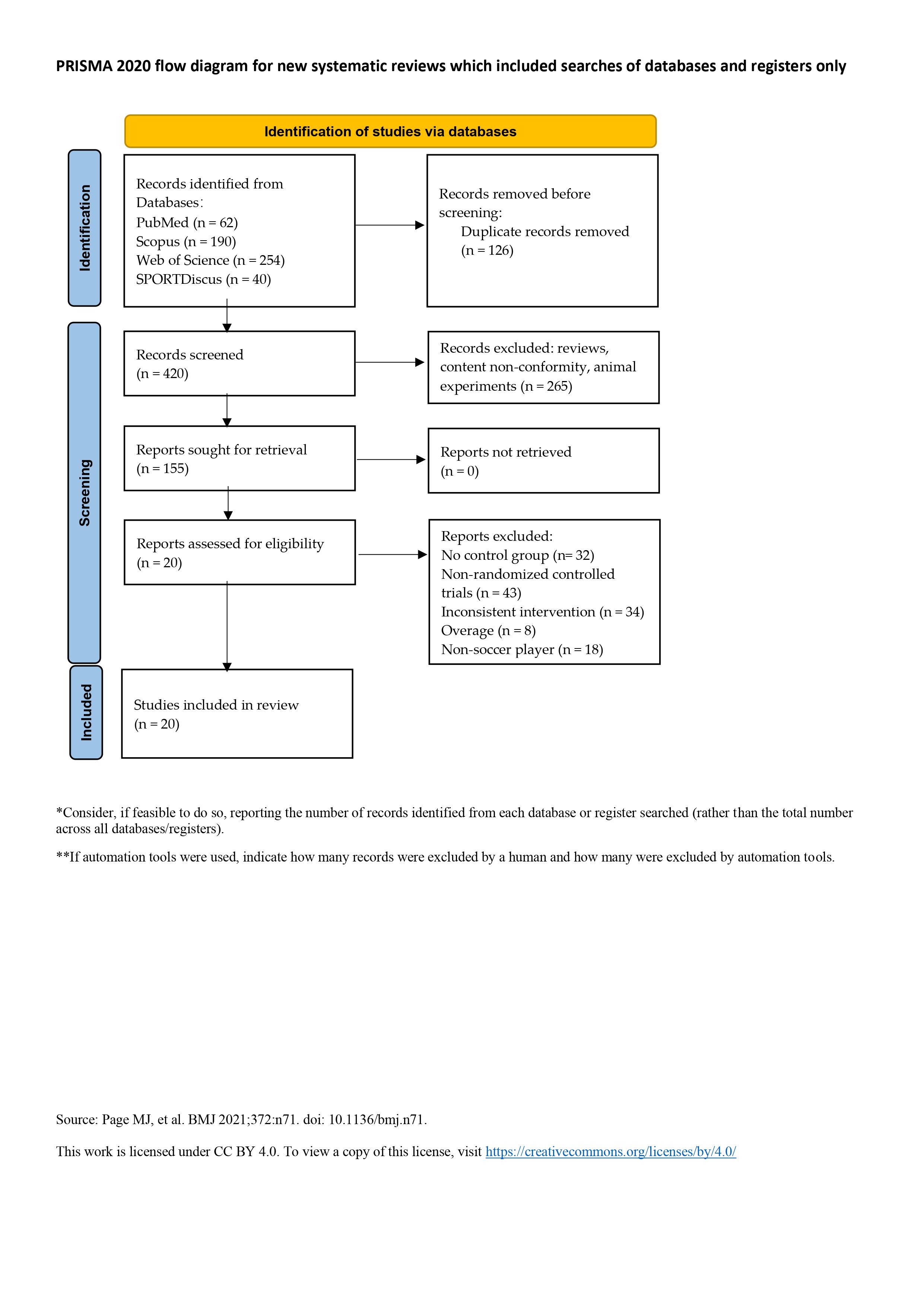 | P3  (L92) |
| Data collection process | 9 | Data from the included studies, as well as participant characteristics, were extracted by one author and compiled in Microsoft Excel. After the data collection was completed, another author verified and confirmed the extracted content. In cases where the extracted information was contentious, a third co-author made the final determination. This study extracted information on the authors of the included studies (first author's name and publication year), characteristics of the study population (gender, age, sample size), and intervention variables (intervention duration, intervention period, intervention frequency). When data were presented in the form of bar charts or error bars, Getadata software was used for data extraction. If data could not be extracted, attempts were made to contact the corresponding authors or reach out via ResearchGate. Should the data remain inaccessible before the publication of the article, the literature in question would be excluded. | P4  (L113-L124) |
| Data items | 10a | \| Outcome categories \| Measure \| \| --- \| --- \| \| Jumping Performance \| Countermovement Jump, CMJ \| \| Squat Jump, SJ \| \| Standing Long Jump, SLJ \| \| Sprinting Performance \| 10M-Sprint, 10M \| \| 20M-Sprint, 20M \| \| 30M-Sprint, 30M \| \| Change of Direction Performance \| Illinois \| \| T-Test \| \| Zig Zag Drill \| | P5  (L134) |
|  | 10b | \| Outcome categories \| Measure \| \| --- \| --- \| \| Jumping Performance \| Countermovement Jump, CMJ \| \| Squat Jump, SJ \| \| Standing Long Jump, SLJ \| \| Sprinting Performance \| 10M-Sprint, 10M \| \| 20M-Sprint, 20M \| \| 30M-Sprint, 30M \| \| Change of Direction Performance \| Illinois \| \| T-Test \| \| Zig Zag Drill \| | P5  (L134) |
| Study risk of bias assessment | 11 | The methodological quality of all included studies in this research was assessed using the PEDro scale (Physiotherapy Evidence Database), with scores ranging from 0 to 10, where a higher score indicates higher quality of the included literature. The quality rating scale for the PEDro scale is categorized as: poor (<4), fair (4-5), good (6-8), and excellent (9-10). The quality of all documents was independently evaluated by two co-authors, with any discrepancies or uncertainties resolved by a third co-author. Publication bias risk was visually inspected using funnel plots and quantified using Egger's test. In the event of publication bias, the missing literature was supplemented using the trim-and-fill method. | P5  (L136-L144) |
| Effect measures | 12 | All data in this study were statistically analyzed using Stata software. The three capabilities assessed in this study—jumping ability, sprinting ability, and change of direction ability—each consist of three different metrics. Therefore, the Standardized Mean Difference (SMD) was selected as the summary effect measure, with the SMD value and its 95% confidence interval presented together. The SMD values can be interpreted as follows: trivial (SMD < 0.20), small (0.20 ≤ SMD < 0.50), moderate (0.50 ≤ SMD < 0.80), and large (SMD ≥ 0.80)[14]. Initially, the data included in this study were the baseline and post-test mean values and standard deviations for both the experimental and control groups. However, to better evaluate the effects between the two groups after the experimental intervention, we artificially transformed the data into change scores and standard deviations, with the calculation formula illustrated in Figure 2[15]. The I2 statistic was used to assess the heterogeneity of the studies; when the I2 value is less than 25%, the heterogeneity can be considered negligible, and a fixed-effect model was used for data analysis. When the I2 value is between 25% and 75%, the studies are considered to have moderate heterogeneity, and a random-effects model was used for data analysis. When the I2 value exceeds 75%, indicating high heterogeneity, a random-effects model was also employed for data analysis[16]. A P-value of less than 0.05 was considered to indicate statistical significance. | P5  (L146-L165) |
| Synthesis methods | 13a | Studies included should involve participants engaging in plyometric training either solely or in conjunction with their regular soccer training. Studies where participants undertake additional training regimens, such as resistance training or high-intensity interval training, alongside plyometric training. | P3  (L111) |
|  | 13b | After the data collection was completed, another author verified and confirmed the extracted content. | P3  (L13-L124) |
|  | 13c | Not applicable | Not applicable |
|  | 13d | When the I2 value is between 25% and 75%, the studies are considered to have moderate heterogeneity, and a random-effects model was used for data analysis. When the I2 value exceeds 75%, indicating high heterogeneity, a random-effects model was also employed for data analysis | P5  (L159-L163) |
|  | 13e | When the I2 value is between 25% and 75%, the studies are considered to have moderate heterogeneity, and a random-effects model was used for data analysis. When the I2 value exceeds 75%, indicating high heterogeneity, a random-effects model was also employed for data analysi | P5  (L159-L163) |
|  | 13f | Not applicable | Not applicable |
| Reporting bias assessment | 14 | The average PEDro scale score of all included studies was 7.55, with the lowest study scoring 7, indicating "good" methodological quality, and four studies scoring 9, indicating "excellent" quality. The funnel plot is depicted in Figure 2. Visual inspection could not quantify publication bias, hence Egger's test was employed for a quantitative analysis of publication bias. The Egger's test revealed the presence of publication bias for jumping ability (t=2.93, p=0.005) and change of direction ability (t=-2.42, p=0.032), while no publication bias was detected for sprinting ability (p=0.059). The trim-and-fill method was applied to address the identified publication bias. After the adjustment, two additional studies were imputed for jumping ability, and the final results remained consistent with the original findings (p=0.000). One additional study was imputed for change of direction ability, and the final results were also consistent with the original findings (p=0.000). These findings suggest that, despite the presence of publication bias, the original outcomes remain robust. | P6  L177-L189 |
| Certainty assessment | 15 | the Standardized Mean Difference (SMD) was selected as the summary effect measure, with the SMD value and its 95% confidence interval presented together. The SMD values can be interpreted as follows: trivial (SMD < 0.20), small (0.20 ≤ SMD < 0.50), moderate (0.50 ≤ SMD < 0.80), and large (SMD ≥ 0.80) | P5  (L150-L152) |
| **RESULTS** | | |  |
| Study selection | 16a | A total of 20 studies were included in this research, encompassing 28 sets of randomized controlled trials. The overall sample size was 796 participants, all of whom were adolescent soccer players (aged 10 to 18.99 years). | P5  (L168-170) |
|  | 16b | When data were presented in the form of bar charts or error bars, Getadata software was used for data extraction. If data could not be extracted, attempts were made to contact the corresponding authors or reach out via ResearchGate. Should the data remain inaccessible before the publication of the article, the literature in question would be excluded. | P4  (L120-124) |
| Study characteristics | 17 | A total of 20 studies were included in this research, encompassing 28 sets of randomized controlled trials. The overall sample size was 796 participants, all of whom were adolescent soccer players (aged 10 to 18.99 years). | P5  (L168-170) |
| Risk of bias in studies | 18 | The methodological quality of all included studies in this research was assessed using the PEDro scale (Physiotherapy Evidence Database), with scores ranging from 0 to 10, where a higher score indicates higher quality of the included literature. The quality rating scale for the PEDro scale is categorized as: poor (<4), fair (4-5), good (6-8), and excellent (9-10). The quality of all documents was independently evaluated by two co-authors, with any discrepancies or uncertainties resolved by a third co-author. Publication bias risk was visually inspected using funnel plots and quantified using Egger's test. In the event of publication bias, the missing literature was supplemented using the trim-and-fill method. | P5  (L136-L144) |
| Results of individual studies | 19 | A total of 20 studies were included in this research, encompassing 28 sets of randomized controlled trials. The overall sample size was 796 participants, all of whom were adolescent soccer players (aged 10 to 18.99 years). | P5  (L168-170) |
| Results of syntheses | 20a | The average PEDro scale score of all included studies was 7.55, with the lowest study scoring 7, indicating "good" methodological quality, and four studies scoring 9, indicating "excellent" quality. The funnel plot is depicted in Figure 2. Visual inspection could not quantify publication bias, hence Egger's test was employed for a quantitative analysis of publication bias. The Egger's test revealed the presence of publication bias for jumping ability (t=2.93, p=0.005) and change of direction ability (t=-2.42, p=0.032), while no publication bias was detected for sprinting ability (p=0.059). The trim-and-fill method was applied to address the identified publication bias. After the adjustment, two additional studies were imputed for jumping ability, and the final results remained consistent with the original findings (p=0.000). | P6  (L177-L188) |
|  | 20b | The findings of the study demonstrated a positive effect of plyometric training on the jumping ability of adolescent soccer players (SMD=0.76, [95%CI: 0.59, 0.93], p<0.001). Subgroup analysis revealed that plyometric training positively influenced all three jumping ability indicators: CMJ (SMD=0.80, [95%CI: 0.55, 1.06], p<0.001), SJ (SMD=0.63, [95%CI: 0.30, 0.95], p<0.001), and SLJ (SMD=0.84, [95%CI: 0.54, 1.14], p<0.001). | P11  (L205-L210) |
|  | 20c | This study incorporated data from 47 sets of randomized controlled trials, involving 1,263 participants, and encompassed three jumping ability indicators: CMJ, squat jump SJ, and SLJ. These indicators were utilized to assess the impact of plyometric training on the lower limb jumping ability of adolescent soccer players (Figure 3). The findings of the study demonstrated a positive effect of plyometric training on the jumping ability of adolescent soccer players (SMD=0.76, [95%CI: 0.59, 0.93], p<0.001). Subgroup analysis revealed that plyometric training positively influenced all three jumping ability indicators: CMJ (SMD=0.80, [95%CI: 0.55, 1.06], p<0.001), SJ (SMD=0.63, [95%CI: 0.30, 0.95], p<0.001), and SLJ (SMD=0.84, [95%CI: 0.54, 1.14], p<0.001). | P11  (L204-L211) |
|  | 20d | The Egger's test revealed the presence of publication bias for jumping ability (t=2.93, p=0.005) and change of direction ability (t=-2.42, p=0.032), while no publication bias was detected for sprinting ability (p=0.059). The trim-and-fill method was applied to address the identified publication bias. After the adjustment, two additional studies were imputed for jumping ability, and the final results remained consistent with the original findings (p=0.000). One additional study was imputed for change of direction ability, and the final results were also consistent with the original findings (p=0.000). These findings suggest that, despite the presence of publication bias, the original outcomes remain robust. | P6  (L181-L189) |
| Reporting biases | 21 | The Egger's test revealed the presence of publication bias for jumping ability (t=2.93, p=0.005) and change of direction ability (t=-2.42, p=0.032), while no publication bias was detected for sprinting ability (p=0.059). The trim-and-fill method was applied to address the identified publication bias. After the adjustment, two additional studies were imputed for jumping ability, and the final results remained consistent with the original findings (p=0.000). One additional study was imputed for change of direction ability, and the final results were also consistent with the original findings (p=0.000). These findings suggest that, despite the presence of publication bias, the original outcomes remain robust. | P6  (L181-L189) |
| Certainty of evidence | 22 | The results of the study indicate that plyometric training has a positive effect on the sprinting ability of adolescent soccer players (SMD=-0.45, [95%CI: -0.57, -0.32], p<0.001). Subgroup analysis revealed that plyometric training positively influenced all three sprinting indicators: 10-meter sprint (SMD=-0.57, [95%CI: -0.82, -0.33], p<0.001), 20-meter sprint (SMD=-0.37, [95%CI: -0.54, -0.21], p<0.001), and 30-meter sprint (SMD=-0.50, [95%CI: -0.85, -0.15], p=0.005). | P12  (L221-L227) |
| **DISCUSSION** | | |  |
| Discussion | 23a | This systematic review and meta-analysis aimed to evaluate whether plyometric training is more effective than conventional soccer training in enhancing the jumping, sprinting, and change of direction abilities of adolescent soccer players. The existing evidence suggests that adolescent soccer players derive significant benefits from plyometric training. Specifically, when conventional soccer training is combined with a well-designed and supervised plyometric training program, adolescent athletes may experience a more substantial improvement in sport performance compared to those who engage solely in soccer practice and matches. | P13-P14  (L251-L258) |
|  | 23b | In this study, we have utilized the maturation categorization standards established by the WHO to delineate the stages of maturation in adolescents. While this approach demonstrates a strong association with individual biological maturity, it is important to acknowledge the potential for bias. | P15  (L382-L388) |
|  | 23c | The subgroup analysis of this study revealed no significant improvements in the performance of adolescent soccer players in the zigzag run and T-test. One possible reason is the limited number of studies included, resulting in a small sample size for the pooled analysis. Additionally, all included studies focused on pre-adolescent athletes. | P15  (L359-L363) |
|  | 23d | Not applicable |  |
| **OTHER INFORMATION** | | |  |
| Registration and protocol | 24a | The study has been registered in the International Prospective Register of Systematic Reviews (PROSPERO: CRD42024579445 ). | P17  (L404-L405) |
|  | 24b | International Prospective Register of Systematic Reviews | P17  (L404-L405) |
|  | 24c | The study has been registered in the International Prospective Register of Systematic Reviews (PROSPERO: CRD42024579445 ). | P17  (L404-L405) |
| Support | 25 | Non-financial support for the review | P17  (L415-L416) |
| Competing interests | 26 | All authors declare that they have no conflict of interest. | P17  (L413) |
| Availability of data, code and other materials | 27 | The datasets used and/or analysed during the current study are available from the corresponding author on reasonable request. | P17  (L409-L411) |

*From:*  Page MJ, McKenzie JE, Bossuyt PM, Boutron I, Hoffmann TC, Mulrow CD, et al. The PRISMA 2020 statement: an updated guideline for reporting systematic reviews. BMJ 2021;372:n71. doi: 10.1136/bmj.n71

For more information, visit: <http://www.prisma-statement.org/>
