# Supplementary material for: Effects of plyometric training on jump, sprint, and change of direction performance in adolescent soccer player: A systematic review with meta-analysis
Source: PLoS One. 2025 Apr 29;20(4):e0319548. doi: 10.1371/journal.pone.0319548 (PMC12040276; doi:10.1371/journal.pone.0319548)
Supplement: Attachment — Submitted filename: PONE-D-24-38516_peer review.pdf [file pone.0319548.s004.pdf]

General comments

The aim of this review was to systematically evaluate the comprehensive effects of plyometric training on adolescent soccer players' jumping, sprinting, and change-of direction abilities, with the intention of providing an empirical basis for coaches and athletes to optimize training programs and enhance competitive performance. This is a study on an interesting research topic, utilizing a systematic review design; however, the methodological procedures for constructing the systematic review, as well as the information extraction process, must be critically improved.

Specific Comments

Please, consider the section-by-section revisions:

- **Introduction:** The authors specify the Chen et al. study, where they report CMJ and 20-meter sprint performance, but plyometric training is something broader. There are methods such as DSI (https://doi.org/10.3389/fspor.2024.1282214), RSI (https://peerj.com/articles/15609/) that should be clarified. Also, field-based tests should be expanded much further to 20 metres (https://link.springer.com/book/10.1007/978-3-031-03895-2), not neglecting the methodological differences between agility and COD. The introduction should end with the objective (and possibly the study hypotheses), never with concluding remarks (lines 68-75). Please rephrase.

- **Methods:**
    - **Literature strategy:** The literature strategy should be registered on a platform such as INPLASY or PROSPERO. Please register and add the code in this subsection. Also, the search strategy is much more than keywords and Boolean operators. This section should report how many authors conducted the search, which intra- and inter-reliability. Also, the author makes no mention of the PRISMA statement, although he reports the flowchart and uses the PICO approach. Please clarify this point (see: https://peerj.com/articles/14381/)
    - **Eligibility Criteria**: The description of the PRISMA and PICOS strategy must be provided beforehand in the search strategy section. The inclusion and exclusion criteria are shown in Table 1.
    - **Data Extraction:** Couldn't this incur a selection bias? For example, you used CMJ and SJ, why don't you evaluate the horizontal jump? Are the t-test, Zig Zag Drill and Illinois test the most commonly used tests to assess COD? Are the 10, 20 and 30 metre sprint tests suitable for all tests?

- **Methodological Quality and Risk of Bias:** The PEDro scale was well applied. I only recommend that you add an analysis of the risk of bias.
- **Statistical Analysis:** This section is well described, but you should clarify the legend in figure 2 (formula).

- **Results:** Some adjustments should be considered. In the Methodological Quality and Risk of Bias Assessment subsection, some statistical tests were not included in the methodology. The presentation of the Forest Plot is clear and precise (figures 3 to 5). However, there are some independent variables presented that are neither reported in the methodology nor discussed. For example: early, late, before, after, fixed, OPT, 4-12 weeks, pre to post-PHV.

- **Discussion:** Following on from the previous comment, the authors should include in the discussion the independent variables they considered for the meta-analysis. The contexts in which they are analysed have yet to be described, not least because there are specificities that must be taken into account in each sport. Also, the limitations section should report on future prospects and practical applications. I have some difficulty understanding objective practical applications for this meta-analysis with regard to Stregnth and Conditioning (please see: Branquinho, L., Ferraz, R., & Marques, M. C. (2021). 5-a-Side Game as a Tool for the Coach in Soccer Training. Strength & Conditioning Journal, 43(5), 96-108.

- **References:** An upgrade should be considered to expand the practical applications and analysis contexts of each study.
