# Supplementary material for: Effects of plyometric training on jump, sprint, and change of direction performance in adolescent soccer player: A systematic review with meta-analysis
Source: PLoS One. 2025 Apr 29;20(4):e0319548. doi: 10.1371/journal.pone.0319548 (PMC12040276; doi:10.1371/journal.pone.0319548)
Supplement: Attachment — Submitted filename: Response to Reviewer2.docx [file pone.0319548.s007.docx]

Dear Reviewer 2,

First and foremost, we sincerely thank you for taking the time to review our manuscript titled "Enhancement of Jump, Sprint, and Change of Direction Abilities: The Efficacy of Plyometric Training in Adolescent Soccer Training—A Systematic Review and Meta-Analysis." Your detailed and constructive comments have been instrumental in refining our work, and we have made significant revisions based on your valuable feedback.

We deeply appreciate your insightful suggestions, which have greatly enhanced the clarity, rigor, and coherence of our paper. We have carefully addressed each point you raised and have revised our manuscript to ensure these changes thoroughly respond to your concerns, presenting our research findings more accurately and persuasively.

Guided by your expertise, we have made the following key improvements to our manuscript:

**Response to comment:**

**• Introduction:**

The authors specify the Chen et al. study, where they report CMJ and 20-meter sprint performance, but plyometric training is something broader. There are methods such as DSI ([https://doi.org/10.3389/fspor.2024.1282214](https://doi.org/10.3389/fspor.2024.1282214#inbox/_blank)), RSI ([https://peerj.com/articles/15609/](https://peerj.com/articles/15609/#inbox/_blank)) that should be clarified. Also, field-based tests should be expanded much further to 20 metres ([https://link.springer.com/book/10.1007/978-3-031-03895-2](https://link.springer.com/book/10.1007/978-3-031-03895-2#inbox/_blank)), not neglecting the methodological differences between agility and COD. The introduction should end with the objective (and possibly the study hypotheses), never with concluding remarks (lines 68-75). Please rephrase.

**Response:**

Thank you for your valuable suggestions regarding the introduction section. Based on your feedback, we have made comprehensive adjustments to present the application of plyometric training in adolescent soccer players more systematically and clearly. The specific revisions are as follows: Firstly, we expanded the discussion on plyometric training’s role in multi-level speed qualities (such as acceleration and maximum speed) to more comprehensively illustrate its application in the development of speed among adolescents. Additionally, we provided a detailed description of change-of-direction ability. As per your suggestion, we also included a discussion on RSI; however, we did not find specific studies addressing the effects of plyometric training on DSI during our search, and thus did not elaborate on this aspect in the introduction.

Finally, the introduction now concludes with a clear statement of the research objectives, avoiding any summary statements, to better align with your expectations.

Thank you for your guidance, and we look forward to your further feedback!

**• Methods:**

**Response to comment:**

o Literature strategy: The literature strategy should be registered on a platform such as INPLASY or PROSPERO. Please register and add the code in this subsection. Also, the search strategy is much more than keywords and Boolean operators. This section should report how many authors conducted the search, which intra- and inter-reliability. Also, the author makes no mention of the PRISMA statement, although he reports the flowchart and uses the PICO approach. Please clarify this point (see: [https://peerj.com/articles/14381/](https://peerj.com/articles/14381/#inbox/_blank))

Thank you for your valuable suggestions on our study. We have carefully reviewed and addressed each of your comments to enhance the quality and rigor of our research report. Our responses to your specific suggestions are as follows:

**Response:**

1. **PROSPERO Registration Number**
   In accordance with your suggestion, we have added the PROSPERO registration number in the **Literature Search** section to ensure transparency and methodological rigor.
2. **Literature Search and Screening Process**
   Regarding the literature search and screening process, we have provided detailed information in the manuscript. The literature search and screening were conducted independently by two reviewers (TJ and RZ). In cases of disagreement, a third reviewer (XW) provided the final decision to ensure consistency in the selection process.
3. **Adherence to the PRISMA Statement**

Following your recommendation, we have revised the manuscript to clarify our adherence to the PRISMA statement. Additionally, we have included relevant references to further improve the description of the process.

We sincerely appreciate your insightful comments and guidance, which have significantly contributed to improving the quality of our research report.

**o Eligibility Criteria:**

**Response to comment:**

The description of the PRISMA and PICOS strategy must be provided beforehand in the search strategy section. The inclusion and exclusion criteria are shown in Table 1.

**Response:**

Thank you for your valuable suggestions on our study. In accordance with your recommendations, we have added a detailed description of the PRISMA and PICOS strategies in the search strategy section to ensure clarity and methodological rigor. We sincerely appreciate your review and guidance, which have helped us further improve the quality of our research report.

**o Data Extraction:**

**Response to comment:**

Couldn't this incur a selection bias? For example, you used CMJ and SJ, why don't you evaluate the horizontal jump? Are the t-test, Zig Zag Drill and Illinois test the most commonly used tests to assess COD? Are the 10, 20 and 30 metre sprint tests suitable for all tests?

**Response:**

Thank you for your valuable suggestions on our study. We appreciate the detailed feedback, and we have carefully considered each of your points regarding data extraction and the assessments used.

1. **Horizontal Jump Assessment**
   Regarding the horizontal jump assessment, we have included the Standing Long Jump (SLJ) as a measure of horizontal jump performance, as indicated in Table 2 of the original manuscript. The SLJ was selected due to its widespread application and its effectiveness in assessing lower limb explosive power, particularly in adolescent soccer players. Additionally, SLJ is a commonly used indicator in the studies included, making it a suitable and representative measure of horizontal jump performance for this population.
2. **Tests for Assessing Change of Direction (COD)**
   For the assessment of COD performance, we selected the T-Test, Zig Zag Drill, and Illinois Test. These tests are widely used in the existing literature, particularly in studies focusing on adolescent soccer players. They have demonstrated good reliability and validity and are effective for evaluating COD ability^1,2^. We also recognize that there are alternative tests for COD, such as the 5-0-5 Agility Test and the Pro-Agility Shuttle; however, we selected the T-Test, Zig Zag Drill, and Illinois Test because they are the most commonly used COD assessments in the literature included in this study. These tests encompass multiple movement patterns involved in COD (e.g., acceleration, deceleration, and directional changes), providing a comprehensive reflection of COD ability in soccer players. In future studies, we may consider including additional types of COD tests to further enhance the evaluation of athletes' change of direction performance.
3. **Suitability of the 10, 20, and 30 Metre Sprint Tests**

Concerning the suitability of the 10, 20, and 30 metre sprint tests, we chose these distances because they are widely used in the literature to assess sprint performance in soccer players and effectively reflect performance at various stages of sprinting. Specifically, the 10-metre sprint test is primarily used to evaluate acceleration, while the 20- and 30-metre sprints capture aspects of maximum speed^3^. These distances cover the different phases of sprinting, from initial acceleration to peak speed, making them appropriate for evaluating the sprinting abilities of adolescent soccer players. While we believe these sprint distances sufficiently assess sprint performance, we may consider including additional distances or other types of sprint tests in future studies to further enrich our evaluation of sprinting abilities.

REFERENCES

1.	Kadlubowski, B., Keiner, M., Hartmann, H., Wirth, K. & Frick, U. The relationship between change of direction tests in elite youth soccer players. Sports 7, 111 (2019).

2.	Kutlu, M., Yapıcı, H., Yoncalık, O. & Çelik, S. Comparison of a new test for agility and skill in soccer with other agility tests. J. Hum. Kinet. 33, 143 (2012).

3.	Young, W. et al. The use of sprint tests for assessment of speed qualities of elite australian rules footballers. Int. J. Sports Physiol. Perform. 3 2, 199–206 (2008).

**o Methodological Quality and Risk of Bias:**

**Response to comment:**

The PEDro scale was well applied. I only recommend that you add an analysis of the risk of bias.

**Response:**

Thank you for your valuable comments on our study. Based on your suggestions, we have made improvements in the risk of bias assessment section. In addition to the PEDro scale, we have employed the Cochrane Risk of Bias 2 (ROB2) tool to systematically evaluate each domain of bias across all included studies. The ROB2 assessment results provide an overview of the distribution of bias risk (see Figure 2 and Table 5) and offer a more comprehensive view of potential biases.Thank you once again for your review and guidance, which have helped us improve the quality of our research report.

**o Statistical Analysis:**

**Response to comment:**

This section is well described, but you should clarify the legend in figure 2 (formula).
**Response:**

Thank you for your valuable suggestions on our study. Based on your feedback, we have added a detailed explanation of the formula in the legend of Figure 2, clearly defining each variable and its purpose in the calculation. The revised legend provides the specific use of the formula and definitions of the variables to ensure that readers can better understand the statistical analysis method used in this study.Thank you again for your review and guidance, which have helped us further improve the quality of our research report.

**• Results:**

**Response to comment:**

Some adjustments should be considered. In the Methodological Quality and Risk of Bias Assessment subsection, some statistical tests were not included in the methodology. The presentation of the Forest Plot is clear and precise (figures 3 to 5). However, there are some independent variables presented that are neither reported in the methodology nor discussed. For example: early, late, before, after, fixed, OPT, 4-12 weeks, pre to post-PHV.
**Response:**

Thank you for your attention to the handling of independent variables in our study and for your valuable suggestions. We fully understand the importance of reporting and discussing independent variables in a meta-analysis and greatly value your input. Below is our detailed response and explanation to your concerns.

In this systematic review, our core objective was to assess the impact of plyometric training on the Jump, Sprint, and Change of Direction Abilities (JSCDA) of adolescent soccer players. These three ability indicators are the focus of our research design and data analysis because we believe they are key metrics for assessing athlete performance and are directly related to the effects of training interventions.

During the methodological quality and risk of bias assessment phase, we focused on these three core ability indicators because we aimed to provide a clear and concentrated perspective on evaluating the effects of training interventions. We recognize that including more independent variables could increase the complexity of the analysis and potentially lead to confusion in the results. Given the limited number of studies currently available and the differences in methodology and measurement tools across studies, we decided to focus on the variables that we believe best reflect the effects of training.

We understand that ignoring other variables that may affect the results could limit the comprehensiveness of our study. However, our choice is based on a detailed analysis of the existing literature and data, as well as considerations for the quality and reliability of the study and its outcomes. Our goal is to provide a robust analytical framework to ensure that our conclusions are both reliable and persuasive.

Your discussion on independent variables provides us with a valuable perspective for further deepening our research. We will elaborate in the discussion section on why we chose these three core ability indicators and how this decision helps us more accurately assess the effects of plyometric training. At the same time, we will also propose future research directions in the discussion section, including how to incorporate more independent variables and how these variables might affect the assessment of training effects.

We greatly appreciate your suggestions and are committed to considering these valuable opinions in our future work, with the aim of making our research more comprehensive and in-depth.

**Response to comment:**

• Discussion: Following on from the previous comment, the authors should include in the discussion the independent variables they considered for the meta-analysis. The contexts in which they are analysed have yet to be described, not least because there are specificities that must be taken into account in each sport. Also, the limitations section should report on future prospects and practical applications. I have some difficulty understanding objective practical applications for this meta-analysis with regard to Stregnth and Conditioning (please see: Branquinho, L., Ferraz, R., & Marques, M. C. (2021). 5-a-Side Game as a Tool for the Coach in Soccer Training. Strength & Conditioning Journal, 43(5), 96-108.

**Response:**

Thank you for your constructive feedback on the limitations and practical applications of our meta-analysis. We have carefully revised the limitations section to align with your suggestions and further clarified the study’s practical implications and future directions.

In response, we have expanded the limitations section to emphasize that, while our study reflects an overall trend in plyometric training effectiveness across a wide age range of adolescents, the absence of systematic biological maturity assessments may limit the generalizability of our findings. Biological maturity significantly impacts neuromuscular coordination, tendon stiffness, and hormonal responses, all of which can influence an adolescent's adaptive response to training. Consequently, we recommend that future studies integrate standardized methods—such as Tanner staging or skeletal age evaluation—to better capture the variations in training adaptations across different developmental stages. Such insights would help to tailor training programs more precisely to each stage, potentially optimizing performance outcomes and minimizing injury risk.

For practical applications, we suggest that strength and conditioning coaches systematically incorporate plyometric training into regular soccer training for adolescent athletes. By adhering to training specificity principles and strategically varying the direction and type of SSC movements, coaches can create comprehensive programs that enhance jumping, sprinting, and change-of-direction abilities, which are critical in soccer performance. This targeted approach, informed by biological maturity considerations, could foster more effective athletic development and ensure training is aligned with the athletes' current capabilities.

We appreciate your guidance on these important aspects and hope that our revisions meet your expectations for a clearer and more applicable interpretation of our findings.

**Response to comment:**

• References: An upgrade should be considered to expand the practical applications and analysis contexts of each study.

**Response:**

Thank you very much for your suggestion regarding the references. We have taken your advice into consideration and have made adjustments to our reference list during the revision process.

We have carefully reviewed our references and removed those with weaker evidence to ensure the robustness of our manuscript. Additionally, we have included more references with stronger evidence to further support our findings and arguments.

Regarding the practical applications section, we have also rewritten it to ensure that it is more closely aligned with our research conclusions. This revision aims to provide clearer guidance for practical applications, making the section more relevant and useful for readers seeking to apply our findings in real-world contexts.

We believe these changes will enhance the quality and applicability of our work, and we appreciate your guidance in helping us achieve this.

We hope that our responses and revisions reflect our commitment to excellence and effectively address your insightful comments. We believe these changes have strengthened our manuscript, making it a suitable candidate for publication in *Plos one*.

We welcome any further suggestions you might have and look forward to your final comments.

Sincerely,
