# Supplementary material for: Effects of plyometric training on jump, sprint, and change of direction performance in adolescent soccer player: A systematic review with meta-analysis
Source: PLoS One. 2025 Apr 29;20(4):e0319548. doi: 10.1371/journal.pone.0319548 (PMC12040276; doi:10.1371/journal.pone.0319548)
Supplement: Attachment — Submitted filename: Manuscript Track Changes_Reviewer.docx [file pone.0319548.s006.docx]

**Enhancement of Jump, Sprint, and Change of Direction Abilities: The Efficacy of Plyometric Training in Adolescent Soccer Training—A Systematic Review and Meta-Analysis**

Tianjing Zheng^1, †^, Runzhou Kong^2, †^, Xiaowen Liang^3, †^, Zhilong Huang^4^, Xicai Luo^1^，Xuan Zhang^5^, Yichao Xiao^5,^ *

1 Guangzhou Railway Polytechnic, Guangzhou, China; 2 Guangzhou Sport University, Guangzhou, China; 3 Guangzhou Children's Palace, Guangzhou, China; 4 Zhongshan Torch Polytechnic, Zhongshan, China;5 Liuzhou Institute of Technology, Liuzhou, China † On behalf of the co-first authors; * On behalf of Correspondence

**Abstract**

**Background:** Soccer, as one of the most popular sports globally, requires a high level of physical fitness, particularly in jumping, sprinting, and change-of-direction abilities, which are crucial for success on the field. For adolescent soccer players, who are in a critical period of physical development, effective training methods are essential to enhance these specific skills. Plyometric training, known for improving lower limb explosiveness, has been extensively studied in adult athletes, but its impact on adolescent soccer players in terms of improving these abilities remains insufficiently and systematically assessed.

**Objective:** This study aims to systematically evaluate the potential for improving the comprehensive effects of plyometric training on adolescent soccer players' jumping, sprinting, and change-of-direction abilities, with the intention of providing an empirical basis for coaches and athletes to optimize training programs and enhance competitive performance.

**Methods:** Eligible randomized controlled trials were identified through searches of PubMed, Web of Science, Scopus, and SPORTDiscus databases, focusing solely on published studies. Study quality was assessed using the PEDro scale, and statistical analysis was conducted using Stata software to calculate the standardized mean difference (SMD) and 95% confidence intervals.

**Results:** A total of 20 studies, comprising 28 randomized controlled trials with a total sample size of 796 participants, were included. The results indicated that plyometric training significantly positively affected the jumping ability (SMD=0.76, 95%CI: 0.59, 0.93), sprinting ability (SMD=-0.45, 95%CI: -0.57, -0.32), and change-of-direction ability (SMD=-0.76, 95%CI: -1.04, -0.47) of adolescent soccer players.

**Conclusion:** Plyometric training is an effective training method to enhance key sport performance measures, including jumping, sprinting, and change-of-direction abilities, in adolescent soccer players. Compared to traditional soccer training, a training regimen incorporating PT significantly improves athletes' overall performance in jumping, sprinting, and change-of-direction abilities. Therefore, it is recommended that PT be included in the training of adolescent soccer players to promote their physical and athletic skill development.

**Keywords:** Plyometric Training; Adolescents; Soccer; Jumping.

**Introduction**

Soccer is one of the most popular sports worldwide, particularly among youth, with participation rates steadily increasing across different regions and age groups. This rise in involvement has led to an increased focus on the physical development of youth soccer players^[1]^. In high-level matches, players are frequently required to execute high-intensity actions such as jumping, sprinting, and changing direction—actions that are crucial for creating scoring opportunities^[2]^. Additionally, sprint speed, jump height, and COD (change of direction) speed have been identified as key factors that differentiate soccer players of varying competitive levels^[3]^. Jump height, in particular, seems to correlate closely with a team’s final league ranking over a season^[4]^. Moreover, many key actions in soccer demand not only maximal power output but also completion within extremely short timeframes (<100 milliseconds), such as sprints and COD movements, which are especially critical in contested possession and scoring situations^[5]^.

In youth soccer matches, high-speed sprints account for only 3% of the total distance covered^[6]^, yet they have a significant impact on decisive moments (e.g., gaining possession, scoring, or conceding goals)^[7]^. Notably, COD ability—which refers to changing movement direction without needing to respond immediately to external stimuli—is strongly linked to sprint and jump performance^[8]^. Enhancing these performance metrics could lead to improvements in COD ability^[9]^. Multiple studies have found that elite and sub-elite youth athletes generally demonstrate higher levels of jumping, running, and COD capabilities than non-elite athletes^[5, 10, 11]^. Since adolescence is a critical window for rapid neuromuscular development^[12]^, selecting suitable training methods is essential for the long-term enhancement of physical fitness and competitive potential in youth athletes^[13]^.

Plyometric training has been widely implemented in team sports such as volleyball^[14]^, basketball^[15]^, and soccer^[16]^, and has consistently been shown to improve various components of muscle strength, including vertical jump^[17]^, speed^[18]^, acceleration^[18, 19]^, maximal strength^[19]^, COD ability^[20, 21]^, reactive strength index (RSI)^[22]^, and sport-specific performance^[23, 24]^. This training method leverages the stretch-shortening cycle (SSC)^[25]^, wherein muscles go through eccentric, isometric, and concentric contractions, effectively storing elastic potential energy^[26]^. When muscles quickly transition from a stretched state to a shortened state, this stored energy is released, allowing the muscles to generate explosive force, thereby significantly boosting an athlete’s power^[27, 28]^.

While some research has examined the effects of plyometric training on jumping and sprinting capabilities in youth soccer players, limitations remain. For example, Ramirez-Campillo et al.’s study^[2]^ included adult soccer players up to 23 years of age, lacking a strict focus on youth players. Chen et al.’s study^[12]^ only evaluated CMJ (countermovement jump) and 20-meter sprint, overlooking multi-dimensional aspects of speed development (e.g., acceleration and top speed) and omitting evaluations of various types of jumps and COD abilities. Additionally, existing research still lacks a comprehensive assessment of different types of jumps, sprints, and COD abilities in youth soccer players, limiting our understanding of plyometric training’s potential for neuromuscular adaptation. Therefore, this study aims to systematically assess the overall effects of plyometric training on jumping, sprinting, and COD abilities in youth soccer players.

**Methods**

**Literature Search**

This review is based on the Preferred Reporting Items for Systematic Reviews and Meta-Analyses (PRISMA) checklist^[29]^. The inclusion criteria were defined using the PICOS framework (Population, Intervention, Comparators, Outcomes, and Study Design) ^[30]^. The study has been registered in the International Prospective Register of Systematic Reviews (PROSPERO: CRD42024579445). To comprehensively identify relevant studies, a search was conducted across four major databases—PubMed, Web of Science (all databases), Scopus, and SPORTDiscus—from the inception of each database up to August 7, 2024. Boolean operators (OR, AND) were utilized in conjunction with a series of keywords, which were finalized based on literature reviews, meta-analyses, expert opinions, and the MeSH Database. The literature search and screening process were conducted independently by two reviewers (TJ and RZ). In cases where discrepancies arose, a third reviewer (XW) provided a final decision to ensure consistency. Furthermore, no language restrictions were applied, and grey literature was excluded from the search strategy. The specific search string was as follows: ("Lower limb explosive strength" OR "Explosive strength" OR "explosive force" OR "Explosive power" OR "power" OR "Countermovement jump" OR "CMJ" OR "squat jump" OR "SJ" OR "standing long jump" OR "SLJ" OR "drop jump" OR "DJ" OR "sprint performance" OR "10m" OR "20m" OR "30m" OR "50m" OR "vertical jump" OR "VJ" OR "change of direction" OR "COD") AND ("plyometric" OR "plyometrics" OR "PT" OR "pliometrique" OR "entrainement pliometrique" OR "salto pliome´trico" OR "velocidad") AND ("Adolescent" OR "Adolescents" OR "Adolescence" OR "Teens" OR "Teen" OR "Teenagers" OR "Teenager" OR "You	th" OR "Youths" OR "Female Adolescent" OR "Female Adolescents" OR "Male Adolescent" OR "Male Adolescents" OR "Child" OR "Children") AND ("soccer"). The screening process is depicted in Figure 1.

**Figure 1.** Visual Overview of the Literature Screening and Inclusion Pathway.

**Eligibility Criteria**

The detailed inclusion and exclusion criteria are shown in Table 1.

**Table 1.** Eligibility Criteria for Inclusion and Exclusion of Studies

| Category | Inclusion criteria | Exclusion Criteria |
| --- | --- | --- |
| Population（P） | The study population consists exclusively of adolescent soccer players, both male and female, with the age range defined as 10 to 18.99 years old | Participants who are not healthy or do not fall within the specified age range for adolescent soccer players. |
| Intervention（I） | Studies included should involve participants engaging in plyometric training either solely or in conjunction with their regular soccer training. | Studies where participants undertake additional training regimens, such as resistance training or high-intensity interval training, alongside plyometric training. |
| Comparator（C） | The control group should only engage in soccer-specific training. | Studies lacking a control group. |
| Outcome（O） | Outcome measures should encompass indicators reflective of jumping, sprinting, and change-of-direction capabilities, with studies being eligible for inclusion whether they incorporate all three indicators or focus on a single one. | Studies without baseline data or where the full text is inaccessible. |
| Study design（S） | Randomized controlled trials | Cross-sectional studies, case studies. |

**Data Extraction**

Data from the included studies, as well as participant characteristics, were extracted by one author and compiled in Microsoft Excel. After the data collection was completed, another author verified and confirmed the extracted content. In cases where the extracted information was contentious, a third co-author made the final determination. This study extracted information on the authors of the included studies (first author's name and publication year), characteristics of the study population (gender, age, sample size), and intervention variables (intervention duration, intervention period, intervention frequency). When data were presented in the form of bar charts or error bars, Getadata software was used for data extraction. In the interpretation of effect sizes, positive standardized mean difference (SMD) values reflect improvements in abilities such as jumping. For measures where lower times indicate better performance, such as sprinting and change-of-direction, negative SMD values signify reduced completion times, corresponding to enhanced performance. If data could not be extracted, attempts were made to contact the corresponding authors or reach out via ResearchGate. Should the data remain inaccessible before the publication of the article, the literature in question would be excluded after three attempts to contact the corresponding authors within two weeks. The extracted metrics are detailed in Table 2.

Table 2. Categories and Metrics for Sport Performance Assessment

| Outcome categories | Measure |
| --- | --- |
| Jumping Performance | Countermovement Jump, CMJ |
|  | Squat Jump, SJ |
|  | Standing Long Jump, SLJ |
| Sprinting Performance | 10M-Sprint, 10M |
|  | 20M-Sprint, 20M |
|  | 30M-Sprint, 30M |
| Change of Direction Performance | Illinois |
|  | T-Test |
|  | Zig Zag Drill |

**Methodological Quality and Risk of Bias**

The methodological quality of all included studies in this research was assessed using the PEDro scale (Physiotherapy Evidence Database), with scores ranging from 0 to 10, where a higher score indicates a higher quality of the included literature. The validity and reliability of the PEDro scale have been established previously^[31]^. The quality rating scale for the PEDro scale is categorized as poor (<4), fair (4-5), good (6-8), and excellent (9-10). The quality of all documents was independently evaluated by two co-authors, with any discrepancies or uncertainties resolved by a third co-author. Publication bias risk was visually inspected using funnel plots and quantified using Egger's test. In the event of publication bias, the missing literature was supplemented using the trim-and-fill method, which identifies asymmetry in funnel plots and estimates missing studies to adjust for potential publication bias.

**Statistical Analysis**

All data in this study were statistically analyzed using Stata software. The three capabilities assessed in this study—jumping ability, sprinting ability, and change of direction ability—each consist of three different metrics. Therefore, the Standardized Mean Difference (SMD) was selected as the summary effect measure, with the SMD value and its 95% confidence interval presented together. The SMD values can be interpreted as follows: trivial (SMD < 0.20), small (0.20 ≤ SMD < 0.50), moderate (0.50 ≤ SMD < 0.80), and large (SMD ≥ 0.80)^[32]^. Initially, the data included in this study were the baseline and post-test mean values and standard deviations for both the experimental and control groups. However, to better evaluate the effects between the two groups after the experimental intervention, we artificially transformed the data into change scores and standard deviations, with the calculation formula illustrated in Figure 2^[33]^. The I^2^ statistic was used to assess the heterogeneity of the studies; when the I^2^ value is less than 25%, the heterogeneity can be considered negligible, and a fixed-effect model was used for data analysis. When the I^2^ value is between 25% and 75%, the studies are considered to have moderate heterogeneity, and a random-effects model was used for data analysis. When the I^2^ value exceeds 75%, indicating high heterogeneity, a random-effects model was also employed for data analysis^[34]^. A P-value of less than 0.05 was considered to indicate statistical significance.

Figure 2 Formula for Converting Change Scores and Standard Deviations: This formula calculates the standard deviation of change scores (SD_change) between baseline and final measurements, where SD_baseline and SD_final represent the standard deviations at baseline and final, respectively, and Corr denotes the correlation between them.

**Results**

**Description of the Included Studies**

A total of 20 studies were included in this research, encompassing 28 randomized controlled trials, with each trial representing a different intervention condition or control group within the studies. The overall sample size was 796 participants, all of whom were adolescent soccer players, with the age range of 10 to 18.99 years used as an inclusion criterion across the studies. The intervention for the experimental group consisted of plyometric training, with variations in protocols across studies in terms of exercise type, intensity, duration, and frequency. In some studies, plyometric training was integrated into regular soccer sessions, while in others, it was conducted separately. The control group engaged solely in soccer training without any additional training interventions. The duration of all studies ranged from 6 to 12 weeks, with intervention frequencies varying from one to three times per week (Table 3).

**Methodological Quality and Risk of Bias Assessment**

The average PEDro scale score of all included studies was 7.55, with the lowest study scoring 7, indicating "good" methodological quality, and four studies scoring 9, indicating "excellent" quality (Table 4). Additionally, to provide a more comprehensive assessment of bias risk, we employed the Cochrane Risk of Bias 2 (ROB2) tool to systematically evaluate each domain of bias in the included studies. The results of the ROB2 assessment are shown in Figure 2 and Table 5. Overall, 60% of the studies were rated as low risk of bias, 35% as having some concerns, and 5% as high risk. These additional analyses provide a more complete overview of potential biases across the included studies.

The funnel plot is depicted in Figure 3. Visual inspection of the funnel plot could not quantify publication bias, hence Egger's test, which is used to detect asymmetry in funnel plots and assess the presence of publication bias, was employed for a quantitative analysis. The Egger's test revealed the presence of publication bias for jumping ability (t=2.93, p=0.005) and change of direction ability (t=-2.42, p=0.032), while no publication bias was detected for sprinting ability (p=0.059). The trim-and-fill method was applied to address the identified publication bias. After the adjustment, two additional studies were imputed for jumping ability, and the final results remained consistent with the original findings (p=0.000). One additional study was imputed for change of direction ability, and the final results were also consistent with the original findings (p=0.000). These findings suggest that, despite the presence of publication bias, the original outcomes remain robust.

**Table 3.** Summary of Study Demographics, Training Parameters, and Outcome Measures

| Studies, years | Gender | Age（Years） | | Sample | | Training Duration  (Minutes) | Frequency  (Times per week) | Period  (Weeks) | Measures |
| --- | --- | --- | --- | --- | --- | --- | --- | --- | --- |
|  |  | PT Group | Control Grouo | PT Group | Control Group |  |  |  |  |
| Vera-Assaoka 2019（early） | Male | 11.2±0.8 | 11.5±0.9 | 16 | 16 | 21 | 2 | 7 | CMJ、20M、Illinois |
| Vera-Assaoka 2019（late） | Male | 14.4±1.0 | 14.5±1.1 | 22 | 22 | 21 | 2 | 7 | CMJ、20M、Illinois |
| Sedano 2011 | / | 18.4±1.1 | 18.2±0.9 | 11 | 11 | / | 2 | 10 | CMJ、SJ、10M |
| Sammoud 2022 | Male | 12.7±0.3 | 12.8±0.3 | 11 | 11 | 35-40 | 3 | 12 | 10M、20M、30M |
| Ramirez-Campillo 2020（Before） | Male | 16.9±0.7 | 17.1±0.5 | 12 | 12 | 20 | 2 | 7 | CMJ、SJ、SLJ、20M、Illinois |
| Ramirez-Campillo 2020（After） | Male | 17.1±0.3 | 17.1±0.5 | 14 | 12 | 20 | 2 | 7 | CMJ、SJ、SLJ、20M、Illinois |
| Ramirez-Campillo 2018（FIXED） | Male | 13.9±1.9 | 13.7±1.6 | 25 | 24 | 13 | 2 | 7 | CMJ、20M、Illinois |
| Ramirez-Campillo 2018（OPT） | Male | 13.1±1.7 | 13.7±1.6 | 24 | 24 | 13 | 2 | 7 | CMJ、20M、Illinois |
| Ramirez-Campillo 2019 | Male | 13.2±1.8 | 13.5±1.9 | 19 | 20 | 20 | 2 | 7 | CMJ、20M、Illinois |
| Ramirez-Campillo 2020 | Male | 12.9±1.9 | 12.6±1.8 | 8 | 7 | 10-15 | 2 | 8 | CMJ、SLJ、30M、ZIG ZAG DRILL |
| Padrón-Cabo 2021 | Male | 12.60±0.70 | 12.39±0.56 | 10 | 10 | 20-35 | 2 | 6 | CMJ、SJ、10M、20M |
| Nurper 2014 | Female | 18.3±2.6 | 18.0±2.0 | 9 | 9 | 60 | 1 | 8 | CMJ、SLJ、20M |
| Negra 2020（4 weeks） | Male | 12.7±0.3 | 12.8±0.3 | 11 | 11 | 35-40 | 2 | 4 | CMJ、SJ、SLJ、20M、Illinois |
| Negra 2020（8 weeks） | Male | 12.7±0.12 | 12.8±0.12 | 11 | 11 | 35-40 | 2 | 8 | CMJ、SJ、SLJ、20M、Illinois |
| Negra 2020（12 weeks） | Male | 12.7±0.17 | 12.8±0.17 | 11 | 11 | 35-40 | 2 | 12 | CMJ、SJ、SLJ、20M、Illinois |
| Negra 2020B | Male | 12.7±0.2 | 12.7±0.2 | 13 | 11 | 25-35 | 2 | 8 | 20M、T-TEST |
| Michailidis 2013 | Male | 10.6±0.6 | 10.6±0.5 | 24 | 21 | 20-25 | 2 | 12 | CMJ、SJ、10M、20M、30M |
| Jlid 2019 | Male | 11.8±0.4 | 11.6±0.5 | 14 | 14 | 20-25 | 2 | 8 | CMJ、SJ、T-TEST |
| Hammami 2016 | Male | 15.7±0.2 | 15.8±0.2 | 15 | 13 | 35 | 2 | 8 | 10M、20M、30M |
| Hammami 2019 | Male | 15.7±0.2 | 15.8±0.2 | 14 | 12 | 35 | 2 | 8 | CMJ、SJ |
| Drouzas 2020 | Male | 10.0 ± 0.5 | 10.2 ± 1.6 | 23 | 22 | 15 | 2 | 10 | CMJ、、10M、20M |
| Chtara 2017 | Male | 13.6±0.3 | 13.6±0.3 | 10 | 10 | 20 | 2 | 6 | 10M、30M、ZIG ZAG DRILL |
| Asadi 2018 (Pre-PHV) | Male | 11.5±0.8 | 11.7±0.4 | 10 | 10 | 30-40 | 2 | 6 | CMJ、SLJ、20M |
| Asadi 2018 (Mid-PHV) | Male | 14.0±0.7 | 14.2±0.6 | 10 | 10 | 30-40 | 2 | 6 | CMJ、SLJ、20M |
| Asadi 2018 (Post-PHV) | Male | 16.6±0.6 | 16.2±0.3 | 10 | 10 | 30-40 | 2 | 6 | CMJ、SLJ、20M |
| Sammoud 2024 | Male | 12.7±0.2 | 11.8±0.4 | 13 | 14 | / | 2 | 8 | CMJ、SLJ |
| Liu 2024 （micro） | Male | 16.3±0.6 | 16.3±0.6 | 17 | 17 | 11 | 2 | 8 | CMJ、SLJ、10M |
| Liu 2024 （reg） | Male | 16.3±0.6 | 16.3±0.6 | 17 | 17 | 14 | 2 | 8 | CMJ、SLJ、10M |

Table 4 Quality Assessment of All Included Studies Based on the PEDro Scale

| Studies | PEDro Scale Items* | | | | | | | | | | | PEDro Score |
| --- | --- | --- | --- | --- | --- | --- | --- | --- | --- | --- | --- | --- |
|  | 1 | 2 | 3 | 4 | 5 | 6 | 7 | 8 | 9 | 10 | 11 |  |
| Vera-Assaoka 2019 | 1 | 1 | 1 | 1 | 0 | 0 | 0 | 1 | 1 | 1 | 1 | 8 |
| Sedano 2011 | 1 | 1 | 0 | 1 | 0 | 0 | 0 | 1 | 1 | 1 | 1 | 7 |
| Sammoud 2022 | 1 | 1 | 0 | 1 | 0 | 0 | 0 | 1 | 1 | 1 | 1 | 7 |
| Ramirez-Campillo 2020 | 1 | 1 | 1 | 1 | 1 | 0 | 0 | 1 | 1 | 1 | 1 | 9 |
| Ramirez-Campillo 2018 | 1 | 1 | 1 | 1 | 1 | 0 | 0 | 1 | 1 | 1 | 1 | 9 |
| Ramirez-Campillo 2019 | 1 | 1 | 0 | 1 | 0 | 0 | 0 | 1 | 1 | 1 | 1 | 7 |
| Ramirez-Campillo 2020 | 1 | 1 | 1 | 1 | 1 | 0 | 0 | 1 | 1 | 1 | 1 | 9 |
| Padrón-Cabo 2021 | 1 | 1 | 1 | 1 | 0 | 0 | 0 | 1 | 1 | 1 | 1 | 8 |
| Nurper 2014 | 1 | 1 | 0 | 1 | 0 | 0 | 0 | 1 | 1 | 1 | 1 | 7 |
| Negra 2020 | 1 | 1 | 0 | 1 | 0 | 0 | 0 | 1 | 1 | 1 | 1 | 7 |
| Negra 2020B | 1 | 1 | 0 | 1 | 0 | 0 | 0 | 1 | 1 | 1 | 1 | 7 |
| Michailidis 2013 | 1 | 1 | 0 | 1 | 0 | 0 | 0 | 1 | 1 | 1 | 1 | 7 |
| Jlid 2019 | 1 | 1 | 0 | 1 | 0 | 0 | 0 | 1 | 1 | 1 | 1 | 7 |
| Hammami 2016 | 1 | 1 | 0 | 1 | 0 | 0 | 0 | 1 | 1 | 1 | 1 | 7 |
| Hammami 2019 | 1 | 1 | 0 | 1 | 0 | 0 | 0 | 1 | 1 | 1 | 1 | 7 |
| Drouzas 2020 | 1 | 1 | 0 | 1 | 0 | 0 | 0 | 1 | 1 | 1 | 1 | 7 |
| Chtara 2017 | 1 | 1 | 0 | 1 | 0 | 0 | 0 | 1 | 1 | 1 | 1 | 7 |
| Asadi 2018 | 1 | 1 | 0 | 1 | 0 | 0 | 0 | 1 | 1 | 1 | 1 | 7 |
| Sammoud 2024 | 1 | 1 | 1 | 1 | 0 | 1 | 0 | 1 | 1 | 1 | 1 | 9 |
| Liu 2024 | 1 | 1 | 1 | 1 | 0 | 0 | 0 | 1 | 1 | 1 | 1 | 8 |

**Table 5.** Study level risk of bias assessment using Cochrane risk of bias tool 2.0 for assessing risk of bias of randomized clinical trials.

| nique ID | Study ID | Randomization  process | Deviations from  intended interventions | Missing outcome  data | Measurement of  the outcome | Selection of  the reported result | Overall Bias |
| --- | --- | --- | --- | --- | --- | --- | --- |
| Vera-Assaoka 2019 | 1 | Low | Low | Low | Low | Low | Low |
| Sedano 2011 | 2 | Some concerns | Low | Low | Low | Low | Some concerns |
| Sammoud 2022 | 3 | Some concerns | Low | Low | Low | Low | Some concerns |
| Ramirez-Campillo 2020A | 4 | Low | Low | Low | Low | Low | Low |
| Ramirez-Campillo 2018 | 5 | Low | Low | Low | Low | Low | Low |
| Ramirez-Campillo 2019 | 6 | Some concerns | Low | Low | Low | Low | Some concerns |
| Ramirez-Campillo 2020B | 7 | Low | Low | High | Low | Low | High |
| Padrón-Cabo 2021 | 8 | Low | Low | Low | Low | Low | Low |
| Nurper 2014 | 9 | Some concerns | Low | Low | Low | Low | Some concerns |
| Negra 2020A | 10 | Some concerns | Low | Low | Low | Low | Some concerns |
| Negra 2020B | 11 | Some concerns | Low | Low | Low | Low | Some concerns |
| Michailidis 2013 | 12 | Some concerns | Low | Low | Low | Low | Low |
| Jlid 2019 | 13 | Some concerns | Low | Low | Low | Low | Some concerns |
| Hammami 2016 | 14 | Some concerns | Low | Low | Low | Low | Some concerns |
| Hammami 2019 | 15 | Some concerns | Low | Low | Low | Low | Some concerns |
| Drouzas 2020 | 16 | Some concerns | Low | Low | Low | Low | Some concerns |
| Chtara 2017 | 17 | Some concerns | Low | Low | Low | Low | Some concerns |
| Asadi 2018 | 18 | Some concerns | Low | Low | Low | Low | Some concerns |
| Sammoud 2024 | 19 | Low | Low | Low | Low | Low | Low |
| Liu 2024 | 20 | Low | Low | Low | Low | Low | Low |

**Figure 2.** Risk of bias of randomized clinical trials

Figure 3 Visualization of Publication Bias, Egger's Test, and Trim-and-Fill Adjustment (A: Funnel plot for Jumping Ability; B: Funnel plot for Sprinting Ability; C: Funnel plot for Change of Direction Ability; D: Egger's Test for Jumping Ability; E: Egger's Test for Sprinting Ability; F: Egger's Test for Change of Direction Ability; G: Trim-and-Fill Adjustment for Jumping Ability; H: Trim-and-Fill Adjustment for Change of Direction Ability)

**Meta-analysis Results**

**The Impact of Plyometric Training on the Jumping Ability of Adolescent Soccer Players**

This study incorporated data from 47 randomized controlled trials, involving 1,263 participants, and encompassed three jumping ability indicators: CMJ, SJ, and SLJ. These indicators were utilized to assess the impact of plyometric training on the lower limb jumping ability of adolescent soccer players (Figure 4). The study demonstrated a moderate positive effect of plyometric training on the jumping ability of adolescent soccer players (SMD=0.76, [95%CI: 0.59, 0.93], p<0.001). Planned subgroup analysis revealed that plyometric training positively influenced all three jumping ability indicators: CMJ (SMD=0.80, [95%CI: 0.55, 1.06], p<0.001), SJ (SMD=0.63, [95%CI: 0.30, 0.95], p<0.001), and SLJ (SMD=0.84, [95%CI: 0.54, 1.14], p<0.001).

**Figure 4.** Forest Plot of the effects of Plyometric Training on Jumping Ability in Adolescent Soccer Players

**The Impact of Plyometric Training on the Sprinting Ability of Adolescent Soccer Players**

This study incorporated data from 34 randomized controlled trials, involving 990 participants, and included three sprinting ability indicators: 10-meter sprint, 20-meter sprint, and 30-meter sprint. These indicators were utilized to assess the impact of plyometric training on the sprinting ability of adolescent soccer players(Figure 5). The study found that plyometric training had a small positive effect on the sprinting ability of adolescent soccer players (SMD=-0.45, [95%CI: -0.57, -0.32], p<0.001). Planned subgroup analysis revealed that plyometric training positively influenced all three sprinting indicators: 10-meter sprint (SMD=-0.57, [95%CI: -0.82, -0.33], p<0.001), 20-meter sprint (SMD=-0.37, [95%CI: -0.54, -0.21], p<0.001), and 30-meter sprint (SMD=-0.50, [95%CI: -0.85, -0.15], p=0.005).

**Figure 5.** Forest Plot of the effects of Plyometric Training on Sprinting Ability in Adolescent Soccer Players

**The Impact of Plyometric Training on the Change of Direction Ability of Adolescent Soccer Players**

This study incorporated data from 14 randomized controlled trials, involving 415 participants, and included three change of direction ability indicators: the Illinois agility test, zig-zag run, and T-test. These indicators were utilized to assess the impact of plyometric training on the change of direction ability of adolescent soccer players (Figure 6). The results of the study indicated that plyometric training had a moderate positive effect on the change of direction ability of adolescent soccer players (SMD = -0.76, [95% CI: -1.04, -0.47], p < 0.001). Planned subgroup analysis revealed that plyometric training positively influenced the Illinois agility test (SMD = -0.71, [95% CI: -0.98, -0.43], p < 0.001), but did not significantly impact the zig-zag run (SMD = -0.38, [95% CI: -1.05, 0.29], p = 0.267) or the T-test (SMD = -1.47, [95% CI: -3.36, 0.42], p > 0.127).

**Figure 6.** Forest Plot of the effects of Plyometric Training on Change of Direction Ability in Adolescent Soccer Players.

**Discussion**

This systematic review and meta-analysis aimed to directly compare the effectiveness of plyometric training with conventional soccer training in enhancing the jumping, sprinting, and change of direction abilities of adolescent soccer players. The existing evidence suggests that adolescent soccer players derive significant benefits from plyometric training. Specifically, incorporating more intense and non-soccer-specific training stimuli, such as a well-designed and supervised plyometric training program, into conventional soccer training may lead to a more substantial improvement in sport performance for adolescent athletes compared to those who engage solely in soccer practice and matches.

**Jumping Performance**

The systematic review and meta-analysis results indicated that plyometric training significantly enhances the jumping, sprinting, and change-of-direction abilities in adolescents. These findings are consistent with previous meta-analytic outcomes^[12, 35, 36]^. Early research suggested that jumping, acceleration, and change-of-direction capacities are distinct qualities that should be targeted with specific training regimens^[37]^. However, recent studies have identified significant correlations among these attributes^[38, 39]^, suggesting that training adaptations often positively affect multiple aspects of athletic performance. These attributes share common neuromuscular adaptation mechanisms and biomechanical patterns, facilitating transfer effects across different training objectives.^[35]^. This may suggest that plyometric training can simultaneously improve athletes' jumping, sprinting, and change-of-direction performances through similar neuromuscular adaptations. Specifically, these neuromuscular functional adaptations include an increased neural drive to the agonist’s muscles, alterations in muscle activation strategies, changes in the mechanical properties of the plantar flexor muscle-tendon complex, and modifications in muscle size and/or structure^[40]^. These adaptations enhance the rate of force development (RFD), optimize energy storage and release during dynamic movements, and improve both intermuscular and intramuscular coordination. Taken together, these adaptations enhance power output, which in turn improves sprint acceleration, vertical jump height, and change-of-direction capabilities.

Furthermore, subgroup analysis revealed that plyometric training, when supplemented with regular soccer training, significantly improves the CMJ (ES=0.8) and SLJ (ES=0.84) performance in adolescent soccer players, but only moderately enhances the SJ (ES=0.63). This difference might occur because plyometric exercises primarily enhance the SSC, which does not fully target pure concentric contraction capabilities, as measured in the SJ test. Consequently, plyometric training is more effective in jump tests involving SSC, while its enhancement in pure concentric jump tests is comparatively limited^[20, 41]^. In contrast, resistance training, which involves less SSC, is more effective in improving SJ performance^[42]^. Therefore, the choice of training modality should be carefully considered based on specific athletic performance requirements. Notably, previous research has shown that control groups engaging only in regular soccer training are insufficient to improve the jumping abilities of adolescent soccer players^[19, 23, 24, 43]^. This may be attributed to the relatively low volume and intensity of jump-specific movements in regular soccer training, which fail to provide the necessary neuromuscular stimulus for significant adaptations. Therefore, these findings underscore the necessity of incorporating structured plyometric training into routine soccer training to target explosive power and optimize the SSC specifically.

**Sprint Performance**

In comparison with traditional soccer training, our findings indicated that while plyometric training significantly improves sprinting capabilities, the magnitude of enhancement is relatively modest (ES=0.45), aligning with the studies of Oliver^[35]^ and Chen^[16]^. This effect may be attributed to adaptations within both central and peripheral neural pathways, alongside enhancements in neuromuscular coordination, which collectively improve joint proprioception and sensitivity to motion. The repetitive activation of articular mechanoreceptors near the terminal range of motion in plyometric exercises likely contributes to these adaptations, providing a plausible basis for the observed improvements in sprint performance associated with plyometric training^[44]^. This may be attributed to the specific training responses related to the direction of force generation in plyometric exercises. Prior research has suggested that vertical plyometric training does not enhance the sprint performance of young soccer players^[45, 46]^. This phenomenon may be attributed to the primary focus of vertical plyometric training on vertical force production, which has limited applicability to the horizontal force requirements essential for sprinting. However, in the current speed meta-analysis, plyometric training typically includes vertical and horizontal exercises. Since the training intervention incorporates horizontal stimuli, which are crucial for the generation of horizontal force during the acceleration phase of linear sprinting (i.e., ≤10 meters) ^[45, 47]^, this may increase the likelihood of adaptation^[48]^. Furthermore, the study by Silva^[49]^ and colleagues found that plyometric training did not significantly improve the linear sprinting ability of adolescent athletes participating in team sports. This could be because team sport athletes frequently perform sprinting, acceleration, deceleration, change-of-direction, and jumping movements in their regular training and competitions, which involve SSC activities that overlap significantly with the stimuli in plyometric training, thereby diminishing the marginal benefits of plyometric training for speed enhancement ^[23]^. However, for athletes with lower baseline strength or those whose regular training lacks high-intensity SSC activities, plyometric training may still play an important role. These athletes may achieve more pronounced neuromuscular adaptations through plyometric training, leading to greater improvements in speed, agility, and movement efficiency. On the other hand, isolated soccer practice, which includes frequent short-distance maximal sprints, may contribute to the development of speed. For instance, the research by Padrón-Cabo et al.^[50]^ demonstrated that six weeks of plyometric training combined with soccer training did not provide additional benefits to the sprint performance of adolescent soccer players, with no significant improvements observed compared to conventional training. Similarly, Negra et al^[21]^. conducted an eight-week, twice-weekly plyometric training program for adolescent soccer players, and the results showed that the improvement in the 20-meter sprint performance of the plyometric training group was less than that of the soccer training group. The study by Michailidis et al.^[23]^ indicated that a 12-week combination of plyometric training and conventional soccer training significantly improved performance in initial acceleration, secondary acceleration, and maximum speed phases. Notably, in the conventional soccer training group, significant improvements in initial and secondary acceleration performance were also observed at the end of the experimental period.

Additionally, we observed a greater improvement trend in 10-meter linear sprinting (ES=0.57) following plyometric training, which is consistent with the findings of Ramirez-Campillo et al.^[51]^. This finding is particularly important in soccer, where over 90% of sprints occur within 20 meters, with short sprints of approximately 10 meters being especially frequent during matches^[52]^. This improvement can be further explained by the type of movement. Plyometric exercises typically involve large joint movements and center of mass displacements, such as CMJ and SLJ. These movements are characterized by slow SSC with longer ground contact times (typically exceeding 250 milliseconds). The 10-meter sprint, as an acceleration phase^[53]^, is characterized by a lower center of mass and longer ground contact times, which is similar to the slow SSC in plyometric training. However, as the sprint distance increases, there is a gradual transition to the maximum speed phase ^[53]^.

The force generation mechanism of athletes shifts from a primary reliance on horizontal force to a greater emphasis on vertical force. This shift results in a significant reduction in ground contact time, requiring athletes to generate higher force outputs in extremely short periods and maintain high stiffness in the lower limbs^[54]^. Since the slow SSC of plyometric training does not perfectly align with the rapid, short-duration ground contact characteristics required in the maximum speed phase, its effectiveness may be limited in this phase. Therefore, the mechanical and neuromuscular demands of plyometric training may shift more towards acceleration and COD, rather than maximum sprint speed. Interestingly, the study by Sáez de Villarreal et al.^[36]^ indicated that linear sprinting abilities of greater than 10 meters were better improved in adult athletes, which differs from our findings.

This difference may stem from the distinct muscle-tendon unit (MTU) stiffness between adolescent and adult athletes. There is supporting evidence that leg stiffness is significantly related to maximum speed but not to acceleration^[55]^. With aging, especially in adulthood, tendons and muscle tissues undergo structural changes, including the thickening and more compact alignment of collagen fibers^[56]^. These changes enhance tendon stiffness, allowing for a faster re-concentration of elastic energy and force transfer to the skeleton, which improves the efficiency of rapid SSC^[57, 58]^. However, in slow SSC activities (acceleration phase), the lower MTU stiffness in adolescents may be more conducive to energy storage and release. Hence, adolescents may exhibit better efficiency in slow SSC activities^[59]^. In summary, speed training should be more specific, and coaches can systematically optimize athletes' performance at different speed phases by strategically combining horizontal and vertical jumps, as well as scientifically configuring fast and slow SSC exercises, thereby comprehensively enhancing speed capabilities.

**COD Performance**

Previous studies have found that plyometric training effectively improves the COD ability of adolescent soccer players, which aligns with our meta-analysis results^[20, 46, 60-62]^. We found that plyometric training led to moderate improvements in COD ability for these athletes (ES=0.76)^[8, 62, 63]^. However, previous studies also showed that athletes undergoing only conventional training did not exhibit significant improvements and even experienced declines in COD performance ^[20, 46, 60]^. These findings further support the necessity of incorporating independent plyometric training during the season to enhance key athletic performance ^[20, 46, 60-63]^. The improvement in COD abilities may be associated with the changes in strength development following plyometric training or an increase in lower limb eccentric strength. This enhanced strength enables athletes to decelerate and change direction more rapidly and effectively^[8]^. The subgroup analysis of this study revealed no significant improvements in the performance of adolescent soccer players in the zigzag run and T-test. One possible reason is the limited number of studies included, resulting in a small sample size for the pooled analysis. Additionally, all included studies focused on pre-adolescent athletes. Compared to their mid- or late-adolescent peers, pre-adolescents have an underdeveloped neuromuscular system, lower levels of anabolic hormones, and less advanced neuromuscular coordination^[64]^. These factors may limit their adaptive response to SSC training stimuli, resulting in relatively modest gains. During the post-peak height velocity (POST-PHV) stage, adolescents undergo significant hormonal and neuromuscular changes, including elevated levels of testosterone, growth hormone, and insulin-like growth factor, which promote muscle hypertrophy and shifts in muscle fiber composition^[65, 66]^. These hormonal changes are accompanied by neuromuscular advancements, such as increased recruitment of high-threshold motor units, reduced co-contraction of agonist-antagonist muscle pairs, and improved coordination due to Golgi tendon organ desensitization. Collectively, these adaptations enable POST-PHV adolescents to utilize the elastic energy mechanisms of SSC activities more effectively, enhancing training outcomes^[67, 68]^. Consequently, the lack of these physiological and neural adaptations in pre-adolescents may explain the absence of statistically significant differences observed in the analysis.

The meta-analysis by Asadi et al.^[9]^ further supports this notion, finding that twice-weekly, 7-week, 1,400-repetition moderate-intensity plyometric training is sufficient to cause a significant enhancement in COD abilities, but the adaptive response to plyometric training depends on the maturity status of adolescents. Compared to mid- and late-adolescents (ES = 0.95–0.99), pre-adolescents (ES = 0.68) experience only a modest improvement in COD abilities. However, the study by Vera-Assaoka et al^[60]^ found that after 7 weeks of plyometric training (twice weekly, 60 jumps per session, including two sets of 10 repetitions from heights of 20, 40, and 60 cm), early adolescents showed a moderate improvement in COD speed (ES = 0.9), while late adolescents exhibited only a minor improvement (ES = 0.28). This finding contrasts with the general trend reported in the meta-analysis by Asadi et al.^[9]^, which suggests that mid- and late-adolescents typically show greater adaptive responses to plyometric training. This discrepancy may imply that differences in biological maturity lead to varying adaptive responses to plyometric training intensities among adolescent athletes.^[69]^. It is noteworthy that in previous studies, agility test scores significantly decreased in the control group after conventional soccer training during the season, further emphasizing the value of independent plyometric training in enhancing the COD abilities of young soccer players during the competitive period^[20, 46, 60]^.

**Limitations**

The sample in this study covers a wide age range of adolescents, reflecting the overall trend of improvement in jumping, sprinting, and change-of-direction abilities through plyometric training. Coaches and strength and conditioning trainers may consider systematically incorporating plyometric training into regular soccer training programs to enhance the jumping, sprinting, and change-of-direction capabilities of adolescent athletes. Additionally, it is recommended that trainers adhere to the principle of training specificity by carefully organizing plyometric exercises in various directions and types of SSC movements to comprehensively improve athletic performance in young soccer players.

However, adolescents' adaptive responses are largely influenced by biological maturity, particularly in the development of neuromuscular coordination, tendon stiffness, and hormonal responses. Due to the lack of systematic assessments of biological maturity in the included studies, the results may not fully capture the variability in adaptive responses across different developmental stages, which could impact the interpretation of training effects and the generalizability of the conclusions. Future research should strengthen the assessment of biological maturity using standard methods such as Tanner staging or skeletal age evaluation, to gain a more comprehensive understanding of adolescents' training adaptations. This approach may also support the development of training programs tailored to their developmental stages, optimizing performance and reducing injury risk.

**Conclusion**

This systematic review and meta-analysis provide a comprehensive assessment of the effects of plyometric training on adolescent soccer players, revealing its marked positive impact on enhancing jumping, sprinting, and change-of-direction abilities. The findings indicate that training programs incorporating plyometric exercises significantly improve jumping, sprinting, and change-of-direction abilities compared to traditional soccer training, demonstrating the efficacy of plyometric training as an auxiliary method to enhance the athletic performance of adolescent soccer players.

**Declarations**

**Consent for publication**

All authors agreed to the published version of the manuscript.

**Availability of data and materials**

All relevant data are within the manuscript and its Supporting Information files.

**Competing interests**

All authors declare that they have no conflict of interest.

**Funding**

Guangdong Province Science and Technology Innovation Strategy Special Fund (Grant Number: pdjh2023b0949).

**Authors' contributions**

Tianjing Zheng contributed to the conception and design of the study, performed the data analysis, and drafted the manuscript. Runzhou Kong was responsible for the acquisition of data, as well as drafting and revising the manuscript for intellectual content. Xiaowen Liang participated in the design of the study, performed the statistical analysis, and helped to draft the manuscript. Zhilong Huang and Xuan Zhang contributed to the data collection and interpretation, and was involved in critically revising the manuscript for important intellectual content. Yichao Xiao oversaw the project, provided professional guidance on the inclusion of adolescent soccer players, and contributed to the final editing of the manuscript. Xicai Luo: Provided financial support for this research. All authors have read and approved the final version of the manuscript.

**Reference**

[1]	Hulteen R M, Smith J J, Morgan P J, et al. Global participation in sport and leisure-time physical activities: A systematic review and meta-analysis [J]. Preventive Medicine, 2017, 95: 14-25.

[2]	Ramirez-Campillo R, Castillo D, Raya-González J, et al. Effects of plyometric jump training on jump and sprint performance in young male soccer players: a systematic review and meta-analysis [J]. Sports Medicine, 2020, 50: 2125-43.

[3]	McKay A K, Stellingwerff T, Smith E S, et al. Defining training and performance caliber: a participant classification framework [J]. International Journal of Sports Physiology and Performance, 2021, 17(2): 317-31.

[4]	Arnason A, Sigurdsson S B, Gudmundsson A, et al. Physical fitness, injuries, and team performance in soccer [J]. Medicine & Science in Sports and Exercise, 2004, 36(2): 278-85.

[5]	le Gall F, Carling C, Williams M, et al. Anthropometric and fitness characteristics of international, professional and amateur male graduate soccer players from an elite youth academy [J]. Journal of Science and Medicine in Sport, 2010, 13(1): 90-5.

[6]	Castagna C, D'OTTAVIO S, Abt G. Activity profile of young soccer players during actual match play [J]. Journal of Strength and Conditioning Research, 2003, 17(4): 775-80.

[7]	Faude O, Koch T, Meyer T. Straight sprinting is the most frequent action in goal situations in professional football [J]. Journal of Sports Sciences, 2012, 30(7): 625-31.

[8]	Sheppard J M, Young W B. Agility literature review: Classifications, training and testing [J]. Journal of Sports Sciences, 2006, 24(9): 919-32.

[9]	Asadi A, Arazi H, Ramirez-Campillo R, et al. Influence of maturation stage on agility performance gains after plyometric training: a systematic review and meta-analysis [J]. Journal of Strength and Conditioning Research, 2017, 31(9): 2609-17.

[10]	Vaeyens R, Malina R M, Janssens M, et al. A multidisciplinary selection model for youth soccer: the Ghent Youth Soccer Project [J]. British Journal of Sports Medicine, 2006, 40(11): 928-34.

[11]	Hansen L, Bangsbo J, Twisk J, et al. Development of muscle strength in relation to training level and testosterone in young male soccer players [J]. Journal of Applied Physiology, 1999, 87(3): 1141-7.

[12]	Chen L, Yan R, Xie L, et al. Maturation-Specific Enhancements in Lower Extremity Explosive Strength Following Plyometric Training in Adolescent Soccer Players: A Systematic Review and Meta-Analysis [J]. Heliyon, 2024.

[13]	Meylan C M, Cronin J B, Oliver J L, et al. An evidence-based model of power development in youth soccer [J]. International Journal of Sports Science and Coaching, 2014, 9(5): 1241-64.

[14]	Ramirez-Campillo R, García-de-Alcaraz A, Chaabene H, et al. Effects of plyometric jump training on physical fitness in amateur and professional volleyball: a meta-analysis [J]. Frontiers in Physiology, 2021, 12: 636140.

[15]	Ramirez-Campillo R, García-Hermoso A, Moran J, et al. The effects of plyometric jump training on physical fitness attributes in basketball players: A meta-analysis [J]. Journal of Sport and Health Science, 2022, 11(6): 656-70.

[16]	Chen L, Zhang Z, Huang Z, et al. Meta-analysis of the effects of plyometric training on lower limb explosive strength in adolescent athletes [J]. International Journal of Environmental Research and Public Health, 2023, 20(3): 1849.

[17]	Asadi A, Ramirez-Campillo R, Arazi H, et al. The effects of maturation on jumping ability and sprint adaptations to plyometric training in youth soccer players [J]. Journal of Sports Sciences, 2018, 36(21): 2405-11.

[18]	Kotzamanidis C. Effect of plyometric training on running performance and vertical jumping in prepubertal boys [J]. Journal of Strength and Conditioning Research, 2006, 20(2): 441-5.

[19]	Drouzas V, Katsikas C, Zafeiridis A, et al. Unilateral plyometric training is superior to volume-matched bilateral training for improving strength, speed and power of lower limbs in preadolescent soccer athletes [J]. Journal of Human Kinetics, 2020, 74(1): 161-76.

[20]	Meylan C, Malatesta D. Effects of in-season plyometric training within soccer practice on explosive actions of young players [J]. Journal of Strength and Conditioning Research, 2009, 23(9): 2605-13.

[21]	Negra Y, Chaabene H, Fernandez-Fernandez J, et al. Short-term plyometric jump training improves repeated-sprint ability in prepuberal male soccer players [J]. Journal of Strength and Conditioning Research, 2020, 34(11): 3241-9.

[22]	Rebelo A, Pereira J R, Martinho D V, et al. How to improve the reactive strength index among male athletes? A systematic review with meta-analysis; proceedings of the Healthcare, F, 2022 [C]. MDPI.

[23]	Michailidis Y, Fatouros I G, Primpa E, et al. Plyometrics' trainability in preadolescent soccer athletes [J]. Journal of Strength and Conditioning Research, 2013, 27(1): 38-49.

[24]	Sedano S, Matheu A, Redondo J, et al. Effects of plyometric training on explosive strength, acceleration capacity and kicking speed in young elite soccer players [J]. Journal of Sports Medicine and Physical Fitness, 2011, 51(1): 50.

[25]	Wilk K E, Voight M L, Keirns M A, et al. Stretch-shortening drills for the upper extremities: theory and clinical application [J]. Journal of Orthopaedic and Sports Physical Therapy, 1993, 17(5): 225-39.

[26]	Komi P. Training of muscle strength and power: interaction of neuromotoric, hypertrophic, and mechanical factors [J]. International Journal of Sports Medicine, 1986, 7(S 1): S10-S5.

[27]	Cavagna G A, Dusman B, Margaria R. Positive work done by a previously stretched muscle [J]. Journal of Applied Physiology, 1968, 24(1): 21-32.

[28]	Komi P V, Gollhofer A. Stretch reflexes can have an important role in force enhancement during SSC exercise [J]. Journal of Applied Biomechanics, 1997, 13(4).

[29]	Shamseer L, Moher D, Clarke M, et al. Preferred reporting items for systematic review and meta-analysis protocols (PRISMA-P) 2015: elaboration and explanation [J]. BMJ, 2015, 350: g7647.

[30]	Liberati A, Altman D G, Tetzlaff J, et al. The PRISMA statement for reporting systematic reviews and meta-analyses of studies that evaluate health care interventions: explanation and elaboration [J]. Annals Of Internal Medicine, 2009, 151(4): W-65-W-94.

[31]	de Morton N A. The PEDro scale is a valid measure of the methodological quality of clinical trials: a demographic study [J]. The Australian journal of physiotherapy, 2009, 55(2): 129-33.

[32]	Hedges L V, Olkin I. Statistical methods for meta-analysis [M]. Academic press, 2014.

[33]	Higgins J P, Green S. Cochrane handbook for systematic reviews of interventions [J]. 2008.

[34]	Higgins J P, Thompson S G, Deeks J J, et al. Measuring inconsistency in meta-analyses [J]. BMJ, 2003, 327(7414): 557-60.

[35]	Oliver J L, Ramachandran A K, Singh U, et al. The effects of strength, plyometric and combined training on strength, power and speed characteristics in high-level, highly trained male youth soccer players: a systematic review and meta-analysis [J]. Sports Medicine, 2024, 54(3): 623-43.

[36]	de Villarreal E S, Requena B, Cronin J B. The effects of plyometric training on sprint performance: a meta-analysis [J]. Journal of Strength and Conditioning Research, 2012, 26(2): 575-84.

[37]	Los Arcos A, Mendiguchia J, Yanci J. Specificity of jumping, acceleration and quick change of direction motor abilities in soccer players [J]. Kinesiology, 2017, 49(1).

[38]	Hammami R, Granacher U, Pizzolato F, et al. Associations between Change of Direction, Balance, Speed, and Muscle Power in Prepubescent Soccer Players. J Athl Enhanc 6: 6 [J]. Journal of Athletic Enhancement, 2017, 6: 2.

[39]	Comfort P, Stewart A, Bloom L, et al. Relationships between strength, sprint, and jump performance in well-trained youth soccer players [J]. Journal of Strength and Conditioning Research, 2014, 28(1): 173-7.

[40]	Markovic G, Mikulic P. Neuro-musculoskeletal and performance adaptations to lower-extremity plyometric training [J]. Sports Medicine, 2010, 40: 859-95.

[41]	Diallo O, Dore E, Duche P, et al. Effects of plyometric training followed by a reduced training programme on physical performance in prepubescent soccer players [J]. Journal of Sports Medicine and Physical Fitness, 2001, 41(3): 342.

[42]	Christou M, Smilios I, Sotiropoulos K, et al. Effects of resistance training on the physical capacities of adolescent soccer players [J]. Journal of Strength and Conditioning Research, 2006, 20(4): 783-91.

[43]	Sammoud S, Negra Y, Bouguezzi R, et al. Effects of plyometric jump training on measures of physical fitness and lower-limb asymmetries in prepubertal male soccer players: a randomized controlled trial [J]. BMC Sports Science, Medicine and Rehabilitation, 2024, 16(1): 37.

[44]	Asadi A. Effects of in-season plyometric training on sprint and balance performance in basketball players [J]. Journal of Sports Sciences, 2013, 6(1): 24-7.

[45]	Ramírez-Campillo R, Gallardo F, Henriquez-Olguín C, et al. Effect of vertical, horizontal, and combined plyometric training on explosive, balance, and endurance performance of young soccer players [J]. Journal of Strength and Conditioning Research, 2015, 29(7): 1784-95.

[46]	Ramírez-Campillo R, Meylan C, Alvarez C, et al. Effects of in-season low-volume high-intensity plyometric training on explosive actions and endurance of young soccer players [J]. Journal of Strength and Conditioning Research, 2014, 28(5): 1335-42.

[47]	Morin J-B, Bourdin M, Edouard P, et al. Mechanical determinants of 100-m sprint running performance [J]. European Journal of Applied Physiology, 2012, 112: 3921-30.

[48]	Kawamori N, Nosaka K, Newton R U. Relationships between ground reaction impulse and sprint acceleration performance in team sport athletes [J]. Journal of Strength and Conditioning Research, 2013, 27(3): 568-73.

[49]	Silva A F, Ramirez-Campillo R, Ceylan H İ, et al. Effects of maturation stage on sprinting speed adaptations to plyometric jump training in youth male team sports players: a systematic review and meta-analysis [J]. Open Access Journal of Sports Medicine, 2022: 41-54.

[50]	Pardos-Mainer E, Lozano D, Torrontegui-Duarte M, et al. Effects of strength vs. plyometric training programs on vertical jumping, linear sprint and change of direction speed performance in female soccer players: a systematic review and meta-analysis [J]. International Journal of Environmental Research and Public Health, 2021, 18(2): 401.

[51]	Ramirez-Campillo R, García-Hermoso A, Moran J, et al. The effects of plyometric jump training on physical fitness attributes in basketball players: A meta-analysis [J]. Journal of Sport and Health Science, 2022.

[52]	Haugen T, Tønnessen E, Hisdal J, et al. The role and development of sprinting speed in soccer [J]. International Journal of Sports Physiology and Performance, 2014, 9(3): 432-41.

[53]	Rumpf M C, Cronin J B, Oliver J L, et al. Assessing youth sprint ability–Methodological issues, reliability and performance data [J]. Pediatric Exercise Science, 2011, 23(4): 442-67.

[54]	Meyers R W, Moeskops S, Oliver J L, et al. Lower-limb stiffness and maximal sprint speed in 11–16-year-old boys [J]. Journal of Strength and Conditioning Research, 2019, 33(7): 1987-95.

[55]	Chelly S M, Denis C. Leg power and hopping stiffness: relationship with sprint running performance [J]. Medicine ＆ Science in Sports ＆Exercise, 2001, 33(2): 326-33.

[56]	Kubo K, Kanehisa H, Kawakami Y, et al. Growth changes in the elastic properties of human tendon structures [J]. International Journal of Sports Medicine, 2001, 22(02): 138-43.

[57]	Bojsen-Møller J, Magnusson S P, Rasmussen L R, et al. Muscle performance during maximal isometric and dynamic contractions is influenced by the stiffness of the tendinous structures [J]. Journal of Applied Physiology, 2005, 99(3): 986-94.

[58]	Bret C, Rahmani A, Dufour A, et al. Leg strength and stiffness as ability factors in 100 m sprint running [J]. Journal of Sports Medicine and Physical Fitness, 2002, 42(3): 274.

[59]	Korff T, Horne S L, Cullen S J, et al. Development of lower limb stiffness and its contribution to maximum vertical jumping power during adolescence [J]. Journal of Experimental Biology, 2009, 212(22): 3737-42.

[60]	Vera-Assaoka T, Ramirez-Campillo R, Alvarez C, et al. Effects of maturation on physical fitness adaptations to plyometric drop jump training in male youth soccer players [J]. Journal of Strength and Conditioning Research, 2020, 34(10): 2760-8.

[61]	Ramirez-Campillo R, Alvarez C, Gentil P, et al. Sequencing effects of plyometric training applied before or after regular soccer training on measures of physical fitness in young players [J]. Journal of Strength and Conditioning Research, 2020, 34(7): 1959-66.

[62]	Ramirez-Campillo R, Alvarez C, Gentil P, et al. Inter-individual variability in responses to 7 weeks of plyometric jump training in male youth soccer players [J]. Frontiers in Physiology, 2018, 9: 1156.

[63]	Chtara M, Rouissi M, Haddad M, et al. Specific physical trainability in elite young soccer players: efficiency over 6 weeks’ in-season training [J]. Biology of Sport, 2017, 34(2): 137.

[64]	Myer G D, Kushner A M, Faigenbaum A D, et al. Training the developing brain, part I: cognitive developmental considerations for training youth [J]. Current Sports Medicine Reports, 2013, 12(5): 304-10.

[65]	Philippaerts R M, Vaeyens R, Janssens M, et al. The relationship between peak height velocity and physical performance in youth soccer players [J]. Journal of Sports Sciences, 2006, 24(3): 221-30.

[66]	Arntz F, Mkaouer B, Markov A, et al. Effect of plyometric jump training on skeletal muscle hypertrophy in healthy individuals: a systematic review with multilevel meta-analysis [J]. Frontiers in Physiology, 2022, 13: 888464.

[67]	Taube W, Kullmann N, Leukel C, et al. Differential reflex adaptations following sensorimotor and strength training in young elite athletes [J]. International Journal of Sports Medicine, 2007: 999-1005.

[68]	Radnor J M, Oliver J L, Waugh C M, et al. The influence of growth and maturation on stretch-shortening cycle function in youth [J]. Sports Medicine, 2018, 48: 57-71.

[69]	Asadi A, Arazi H, Young W B, et al. The effects of plyometric training on change-of-direction ability: A meta-analysis [J]. International Journal of Sports Physiology and Performance, 2016, 11(5): 563-73.
