# Supplementary material for: Effects of plyometric training on jump, sprint, and change of direction performance in adolescent soccer player: A systematic review with meta-analysis
Source: PLoS One. 2025 Apr 29;20(4):e0319548. doi: 10.1371/journal.pone.0319548 (PMC12040276; doi:10.1371/journal.pone.0319548)
Supplement: Attachment — Submitted filename: Response_to_Reviewer2_auresp_2.docx [file pone.0319548.s008.docx]

**Dear Reviewer 2,**

We sincerely appreciate your valuable comments and constructive suggestions regarding our manuscript. Your insightful feedback has been instrumental in refining the quality and rigor of our study, as well as in broadening the scope of our discussion. Your comments not only highlight areas for improvement but also provide valuable perspectives that have enhanced the overall clarity, focus, and relevance of our work. We deeply value the time and effort you have dedicated to reviewing our manuscript and providing us with actionable recommendations.

In response, we have carefully addressed each of your suggestions to ensure that our revisions meet the high standards of rigor and precision expected in this field. Your feedback has prompted us to re-evaluate and refine multiple sections of the manuscript, from the title and abstract to the methods, results, and discussion. We have also incorporated additional references and expanded on certain topics to ensure that the manuscript is both comprehensive and focused, while adhering to the predefined scope of our study.

Below, we provide a detailed point-by-point summary of how we have incorporated your suggestions into the manuscript. We hope these revisions address your concerns and further enhance the scientific value and practical applicability of our research.

**1.Response to comment:**

Title: Replace ‘Adolescent’ with ‘Youth’, as it allows a more comprehensive view of the different stages of football player development.

**Response:**

Thank you very much for your valuable feedback and the suggestion to replace the term "Adolescent" with "Youth" in the title to provide a broader perspective on football player development.

We would like to clarify that the study population in our systematic review exclusively consists of adolescent soccer players, both male and female, with the age range defined as 10 to 18.99 years old. Given this specific age range, we believe the term "Adolescent" is more precise and better aligns with our inclusion criteria and the developmental stage of the participants studied.

While "Youth" indeed encompasses a broader age range, including young adults beyond adolescence, using "Adolescent" ensures that the title accurately reflects the focus and scope of the population examined in our review. We hope this explanation clarifies our rationale for retaining this terminology.

We truly value your insightful comments and are happy to discuss this further if needed.

Thank you again for your valuable insights and for helping us refine our work.

**2.Response to comment:**

Present the background more concisely, prioritising 2 sentences: an introductory sentence and a second sentence with the objectives of the study.Change-of-direction (COD) tends to be a commonly used abbreviation in this context (put it in the abstract as you did in the full text). Also, mention what PT means in the first sentence. Pliometric training?
**Response:**

Thank you for your valuable feedback, which has greatly helped us refine our manuscript.

In response to your suggestions, we have made the following revisions to the abstract:

1.The background has been condensed into two sentences for clarity and focus. The revised background now reads:

**Background:** Soccer requires a high level of physical fitness, particularly in jumping, sprinting, and change-of-direction (COD) performance. Plyometric training has been extensively studied in adult athletes, but its effects on these abilities in adolescent soccer players remain insufficiently evaluated.

2.We have consistently used "change-of-direction (COD)" as an abbreviation in the abstract, aligning with its usage in the full text.

3.To ensure clarity and readability, we have decided to use "Plyometric training" in full throughout the abstract instead of introducing the abbreviation "PT".

We hope these changes address your comments effectively. Please let us know if there are any additional suggestions or areas for improvement.

**3.Response to comment:**

In the results, present a qualitative assessment of the magnitude of the effects on SMD.

**Response:**

Thank you for your valuable feedback, particularly your suggestion to include a qualitative assessment of the magnitude of the effects on SMD in the results section.

We have revised the results section to incorporate this qualitative assessment, explicitly describing the effects as "moderate" for jumping ability (SMD=0.76) and COD (SMD=-0.76) and as "small" for sprinting ability (SMD=-0.45). The updated results section now reads:

**Results:** A total of 20 studies, comprising 28 randomized controlled trials with a total sample size of 796 participants, were included. The results indicated that plyometric training significantly improved the jumping ability (SMD=0.76, 95%CI: 0.59, 0.93; moderate effect), sprinting ability (SMD=-0.45, 95%CI: -0.57, -0.32; small effect), and COD (SMD=-0.76, 95%CI: -1.04, -0.47; moderate effect) of adolescent soccer players.

We hope this revision addresses your comment effectively. Please let us know if there are any further adjustments or improvements you would recommend.

**4.Response to comment:**

Introduction: In the last chapter, expand on other indices such as CPPF, DSI, IMF variables (please check: [https://doi.org/10.3389/fspor.2024.1282214](https://doi.org/10.3389/fspor.2024.1282214)). The effect of PT on these variables should be deepened.

**Response:**

Thank you very much for your valuable suggestions, especially regarding the inclusion of a discussion on indices such as CPPF, DSI, and IMF in the last paragraph of the introduction. I have carefully reviewed the article you recommended and reflected on how to better incorporate these aspects into this paper.

After careful consideration, I believe that expanding on these indices in the final paragraph of the introduction may not be entirely appropriate. The primary purpose of the final paragraph in the introduction is to establish the research objectives and highlight the significance of the study. Adding an extended discussion on these indices at this point could potentially deviate from the main focus of the paper and undermine the clarity of the research goals. For this reason, I opted to incorporate and discuss these concepts in the third paragraph instead.

Specifically, in the third paragraph, I emphasized the characteristics of neuromuscular development in adolescents, such as the gradual increase in concentric peak force (CPPF) and isometric maximal force (IMF), which reflects the synergy between strength development and neuromuscular maturation, thereby offering greater potential for training adaptations.

As for the last paragraph of the introduction, I prefer to focus on identifying the research gaps and limitations in existing studies, clearly introducing the research objectives of this study, and maintaining the logical coherence and focus of the paper.

Once again, thank you for your suggestion, which has prompted me to think more deeply about the structure and content of the paper. If you have further suggestions on this adjustment, I would be happy to refine these sections accordingly.

**5.Response to comment:**

The methodology is unimpeachable. I only suggest that you add the authors who selected and extracted the information from the articles, as well as inter-observer reliability. A third author should be considered to resolve discrepancies in the interpretation and extraction of information.

**Response:**

Thank you for your thoughtful feedback and constructive suggestions regarding the methodology. We appreciate your recognition of the methodological rigor and have carefully reviewed your comments on inter-observer reliability and the roles of the authors in data selection and extraction.

In the revised manuscript, we have clarified that the literature search and screening process were conducted independently by two reviewers (TJ and RZ). Any discrepancies were resolved by a third reviewer (XW) to ensure consistency and reliability. Similarly, for data extraction, one author (TJ) initially extracted the information, which was then verified and confirmed by another author (ZL). Disputes regarding the extracted information were also resolved by a third co-author (XW). This process was designed to ensure transparency and inter-observer reliability throughout.

We hope this explanation satisfies your request for additional details. While we have not performed a formal statistical analysis for inter-observer reliability, we believe the current process effectively ensures consistency in data handling. If you feel a formal reliability analysis is necessary, we would be happy to incorporate it in future revisions.

**6.Response to comment:**

Table 2: Why the three field tests were selected to assess Jumping Performance, Sprinting Performance and Change of Direction Performance. What were the criteria for selecting these tests? In addition, it should be made clear whether the included studies should integrate all three performance dimensions (jumping, sprinting or COD) or just one of the three.

**Response:**

Thank you for your valuable feedback and insightful suggestions regarding our study. We appreciate your attention to the details of our methodology, especially concerning the selection of outcome measures for jumping, sprinting, and change-of-direction (COD) capabilities.

We have revised the manuscript to clarify the rationale behind the selection of these field tests. Specifically, we selected these outcome measures based on their established reliability and relevance to the physical demands of soccer. These tests are widely recognized and validated in the literature as effective tools for assessing soccer-specific athletic performance. Additionally, we have clarified that studies were eligible for inclusion if they evaluated at least one of these indicators, rather than requiring all three^2–4^.

Outcome measures were selected to encompass indicators reflective of jumping, sprinting, and change-of-direction capabilities, based on their established reliability and relevance to the physical demands of soccer, with studies being eligible for inclusion if they incorporate at least one of these indicators.

We hope this revision addresses your concerns and provides a clear explanation of our methodology. Please let us know if further elaboration or additional references are needed.

**7.Response to comment:**
In fact the meta analysis is robust, however some RCT studies should have been included: (e.g. [https://doi.org/10.1016/j.gaitpost.2023.06.025](https://doi.org/10.1016/j.gaitpost.2023.06.025) or [https://link.springer.com/article/10.1186/s13102-022-00592-1](https://link.springer.com/article/10.1186/s13102-022-00592-1)). Please consider the previous point so that the meta-analysis actually covers all the relevant research in this area.

**Response:**

Thank you for your valuable suggestions regarding the inclusion of additional studies, particularly those referenced in your feedback. After a thorough evaluation of the two suggested articles, we would like to respectfully explain why these studies are not aligned with the scope and inclusion criteria of our meta-analysis.

1.First Suggested Study
Citation: [https://doi.org/10.1016/j.gaitpost.2023.06.025](https://doi.org/10.1016/j.gaitpost.2023.06.025)
This study combines strength training, plyometric training, and running technique training as a multifaceted intervention. While plyometric training is included as a component, the integration of additional training modalities (strength and running technique) makes it impossible to isolate the independent effects of plyometric training on jumping, sprinting, and change-of-direction (COD) performance. As outlined in our inclusion and exclusion criteria (Table 1), studies combining plyometric training with other interventions were explicitly excluded to minimize heterogeneity and ensure consistency across the included studies. Including this study would undermine the validity of our analysis by introducing variability in training mechanisms and intensity, making direct comparisons between studies challenging.

2.Second Suggested Study
Citation: [https://link.springer.com/article/10.1186/s13102-022-00592-1](https://link.springer.com/article/10.1186/s13102-022-00592-1)
This study evaluates the effects of small-sided games (SSGs) as an intervention, specifically comparing 1v1 and 5v5 formats. While SSGs are valuable for improving physical fitness, tactical performance, and decision-making, they do not meet the criteria for plyometric training, which is characterized by high-intensity explosive exercises focused on leveraging the stretch-shortening cycle (SSC). Including studies on SSGs would introduce heterogeneity in the types of interventions analyzed, as the objectives, intensity, and mechanisms of SSGs differ significantly from those of plyometric training. Furthermore, our meta-analysis explicitly focuses on the effects of plyometric training as a standalone intervention, and not on multifactorial or sport-specific interventions such as SSGs.

Both suggested studies involve combined or alternative training interventions, which do not align with our inclusion criteria. Including them would conflict with the primary goal of our meta-analysis, which is to assess the specific effects of plyometric training on jumping, sprinting, and COD performance. Moreover, the inclusion of interventions with fundamentally different objectives and mechanisms would increase heterogeneity, reducing the reliability and interpretability of the pooled results. Our inclusion criteria were carefully defined to ensure the meta-analysis evaluates the distinct impact of plyometric training alone, providing clear and actionable conclusions for practitioners and researchers.

We sincerely appreciate your thoughtful recommendations and acknowledge the relevance of these studies to broader discussions on soccer training methodologies. However, to maintain the focus and rigor of our meta-analysis, we believe it is essential to adhere strictly to our predefined inclusion criteria. We remain open to further suggestions and welcome any additional feedback to improve the quality and robustness of our work.

Thank you again for your time and effort in reviewing our manuscript.

**8.Response to comment:**
Discussion:
The study considers the effects of PT to improve the comprehensive effects of plyometric training on the jumping, sprinting and change of direction skills of adolescent footballers, with the intention of providing an empirical basis for coaches and athletes to optimise training programmes and improve competitive performance. However, the authors do not clarify how it can influence taking into account field position ([https://peerj.com/articles/15609/](https://peerj.com/articles/15609/)), age group ([https://doi.org/10.3389/fpsyg.2024.1447968](https://doi.org/10.3389/fpsyg.2024.1447968)), contexts or tactical issues ([https://doi.org/10.3389/fpsyg.2024.1447968](https://doi.org/10.3389/fpsyg.2024.1447968)).
Discussion:
As for future recommendations on the control of biological maturation, it would also be interesting to analyse the relationship between plyometric training with jumping, sprinting and COD performance in the context of monitoring training load (please, see: [https://doi.org/10.3389/fphys.2022.832202](https://doi.org/10.3389/fphys.2022.832202)) and well-being ([https://doi.org/10.1371/journal.pone.0225522](https://doi.org/10.1371/journal.pone.0225522)).

**Response:**

Thank you for your insightful comments and constructive suggestions. We sincerely appreciate the time and effort you have taken to review our manuscript, as well as your valuable recommendations for improving the quality and rigor of our study. Following your suggestions, we have carefully revised the manuscript and addressed the specific points you raised.

1.On the Discussion of Field Position, Age Groups, and Tactical Issues:

We fully agree with your suggestion that variables such as field position, age groups, and tactical demands are critical factors that can influence training outcomes in adolescent soccer players. To address this, we have expanded the "Limitations" section of the manuscript to acknowledge the potential impact of these variables. Specifically, we have discussed how these factors not only affect the physical demands placed on athletes but may also modulate their adaptive responses to plyometric training. To provide further context, we have incorporated references to the recommended literature. These additions aim to emphasize the need for future research to explore how plyometric training can be tailored to specific field positions, age groups, and tactical contexts to optimize its effectiveness.

2.On Monitoring Training Load and Well-Being:

Your suggestion to explore the relationship between plyometric training, training load monitoring, and well-being is highly relevant. In response, we have integrated a discussion of these aspects into the "Limitations" section. Specifically, we referenced the suggested studies to highlight the importance of monitoring training load and fatigue as part of plyometric training interventions. While our meta-analysis did not directly address these variables, we recognize their importance in optimizing training strategies, enhancing performance outcomes, and mitigating potential health risks. These considerations have been recommended as key areas for future research.

We hope these revisions address your valuable comments and suggestions. The changes we have made aim to improve the depth and clarity of the discussion, as well as to highlight important areas for future research. Thank you once again for your thoughtful feedback, which has significantly enhanced the quality of our manuscript. We look forward to hearing your thoughts on the revised version.

Revised Limitations Section:

Below is the revised "Limitations" section, reflecting the changes made in response to your suggestions:

Limitations

The sample in this study encompasses a broad age range of adolescents, reflecting the overall improvements in jumping, sprinting, and change-of-direction abilities achieved through plyometric training. Coaches and strength and conditioning trainers may consider systematically incorporating plyometric training into routine soccer training programs to enhance these key performance metrics in adolescent athletes. However, adolescents' adaptive responses are significantly influenced by biological maturity, particularly in the development of neuromuscular coordination, tendon stiffness, and hormonal responses. The lack of systematic assessments of biological maturity in the included studies may limit the ability to fully capture variations in adaptive responses across different developmental stages, potentially affecting the interpretation of training effects and the generalizability of the conclusions. Future research should strengthen assessments of biological maturity using standardized methods such as Tanner staging or skeletal age evaluation to better understand adolescents' training adaptations^5^.

Additionally, the adaptive responses of adolescent athletes may also be modulated by variables such as playing position^6^, age groups^7^, and tactical demands^8^. These variables not only directly impact physical demands but may also influence the effectiveness of training adaptations. Future research should further explore the application of plyometric training in the context of these variables and integrate comprehensive analyses of training load and fatigue monitoring to optimize training strategies, enhance outcomes, and mitigate potential health risks^9,10^.

Reference

1.	Portella, D. L. *et al.* Comparison of force variables and dynamic strength index between age groups in elite young brazilian football goalkeepers. *Front. Sports Act. Living* **6**, (2024).

2.	Faude, O., Koch, T. & Meyer, T. Straight sprinting is the most frequent action in goal situations in professional football. *J. Sports Sci.* **30**, 625–631 (2012).

3.	Slinde, F., Suber, C., Suber, L., Edwén, C. E. & Svantesson, U. Test-retest reliability of three different countermovement jumping tests. *J. Strength Cond. Res.* **22**, 640–644 (2008).

4.	Popowczak, M. *et al.* Are linear speed and jumping ability determinants of change of direction movements in young male soccer players? *J. Sports Sci. Med.* **18**, 109–117 (2019).

5.	Malina, R. M. *et al.* Tanner–whitehouse skeletal ages in male youth soccer players: TW2 or TW3? *Sports Med.* **48**, 991–1008 (2018).

6.	Sampaio, T., Marinho, D., Teixeira, J. E., Oliveira, J. & Morais, J. Clustering U-14 portuguese regional team football players by lower limb strength, power, dynamic balance, speed and change of direction: Understanding the field position factor. *PeerJ* **11**, e15609 (2023).

7.	Teixeira, J. E. *et al.* Classification of recovery states in U15, U17, and U19 sub-elite football players: A machine learning approach. *Front. Psychol.* **15**, (2024).

8.	Teixeira, J. E. *et al.* Integrating physical and tactical factors in football using positional data: a systematic review. *PeerJ* **10**, e14381 (2022).

9.	Teixeira, J. E. *et al.* Effects of chronological age, relative age, and maturation status on accumulated training load and perceived exertion in young sub-elite football players. *Front. Physiol.* **13**, (2022).

10.	Clemente, F. M. *et al.* Variations of training load, monotony, and strain and dose-response relationships with maximal aerobic speed, maximal oxygen uptake, and isokinetic strength in professional soccer players. *PLOS ONE* **14**, e0225522 (2019).
